# Engineering of Specific Single-Module Nonribosomal Peptide Synthetases of the RXP Type for the Production of Defined Peptides

**DOI:** 10.1021/acssynbio.2c00472

**Published:** 2022-12-19

**Authors:** Xiaofeng Cai, Lei Zhao, Helge B. Bode

**Affiliations:** † School of Pharmacy, Tongji Medical College, Huazhong University of Science and Technology, 430030 Wuhan, China; ‡ Molecular Biotechnology, Department of Biosciences, Goethe University Frankfurt, 60438 Frankfurt am Main, Germany; § State Key Laboratory of Bio-organic and Natural Products Chemistry, Shanghai Institute of Organic Chemistry, Chinese Academy of Sciences, 200032 Shanghai, China; ∥ Department of Natural Products in Organismic Interactions, Max-Planck-Institute for Terrestrial Microbiology, 35043 Marburg, Germany; ⊥ Chemical Biology, Department of Chemistry, Philipps University Marburg, 35037 Marburg, Germany; # Senckenberg Gesellschaft für Naturforschung, 60325 Frankfurt, Germany

**Keywords:** rhabdopeptide/xenortide-like peptides, single-module NRPSs, heterologous expression, engineering, module flipping

## Abstract

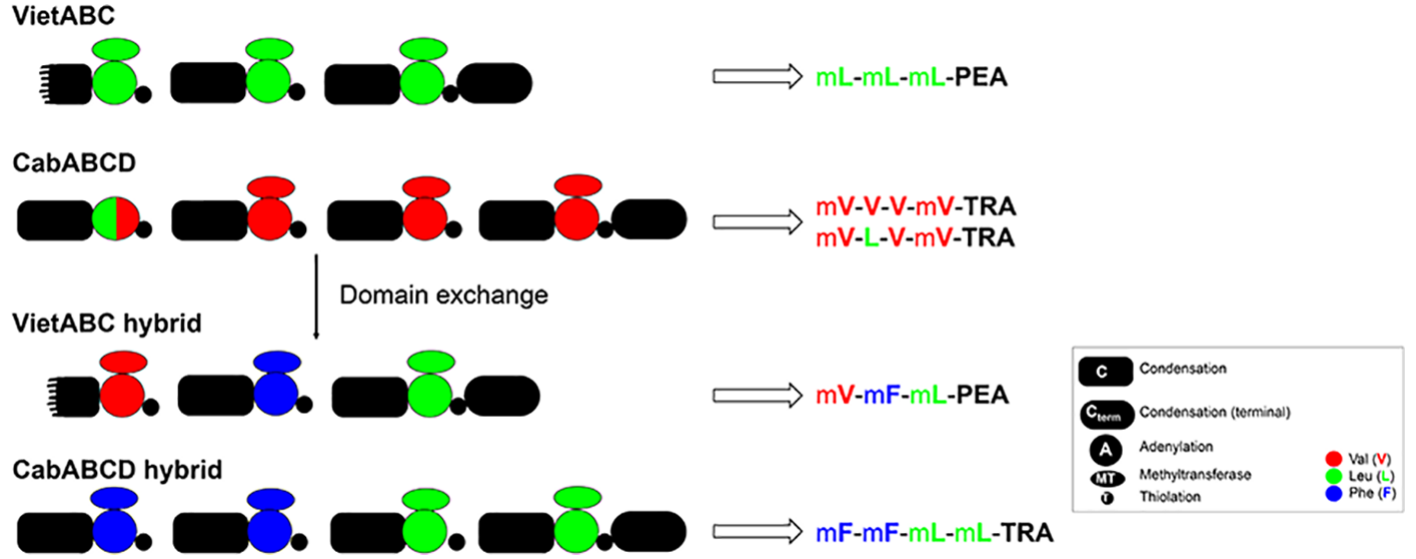

Rhabdopeptide/xenortide-like peptide (RXP) nonribosomal peptide synthetases (NRPSs) derived from entomophathogenic *Xenorhabdus* and *Photorhabdus* bacteria often produce libraries of different peptides varying in amino acid composition, number and degree of methylation, which mainly is a result of promiscuous docking domains (DDs) mediating protein–protein interactions between the different NRPS subunits. In this study, we present two specific RXP-NRPS systems with rather specific DDs that were used as platforms to generate a series of defined RXPs via the exchange of adenylation/methyltransferase (A-MT) domains in the systems followed by heterologous expression in *Escherichia coli*. Additionally, these results suggest that NRPS subunit interaction is not only exclusively dependent on DDs but at least partially also on A domains.

## Introduction

Nonribosomal peptides (NRPs) represent a large family of natural products with diverse bioactivities as antifungals, antibiotics, antitumor agents, siderophores, pigments, and immunosuppressants.^1−6^ NRPs are synthesized by multimodular or monomodular megaenzyme complexes named nonribosomal peptide synthetases (NRPSs).^1−8^ Three types of NRPSs had been classified and comprehensively described previously. Type A NRPSs follow the “collinearity rule”.^3,6^ Each NRPS module is only used once and is responsible for the addition of one specific amino acid into the growing polypeptide chain. The final released linear or cyclic peptides correspond to the sequential organization of NRPSs, such as NRPSs for the biosynthesis of cyclic NRP surfactin from *Bacillus subtilis*(9) and cyclosporin A from *Tolypocladium inflatum*(10) as well as linear NRP kolossin A from *Photorhabdus luminescens*.^11^ Iterative type B NRPSs, in which either a single-module NRPS or a multimodular NRPS can be used multiple times to produce NRPs, consist of repetitive sets of amino acids.^3,6^ The examples are a single-module EntF for the synthesis of enterobactin in *E. coli*(12) and a multimodule GrsB involved in gramicidin S production by *Bacillus brevis*.^13^ Nonlinear type C NRPSs are literally the extended family of iterative NRPSs. The domain organizations are generally unusual and certain domain is inactive but another acts more than once for the assembly of one or multiple NRPs.^3,6^ They can be represented by VibF for the synthesis of siderophore vibriobactin,^14^ more impressive NRPSs involved in yersiniabactin^15−18^ and WS9326A^19^ biosynthesis, respectively. However, with an increasing number of reports on ribosomally independent peptides that are assembled by atypical NRPS systems, the Hertweck group recognized the problem of previous NRPS nomenclature and recently proposed a new framework to classify ribosome-independent peptide synthetases. In this framework, NRPSs as a large family of ribosome-independent peptide synthetases have been divided into three types: type I are modular NRPSs corresponding to the early classification of type A and type B NRPSs; type II are derived from type I, which consist of fully or partially freestanding NRPS domains matching with the early description of part of type C NRPSs; and type III are built from freestanding NRPS domains, catalyzing amide bond formation by noncanonical biocatalysts instead of the condensation (C) domain.^20^

Rhabdopeptide/xenortide-like peptides (RXPs) are nonribosomally made linear peptides widely found in entomophathogenic bacteria of the genera *Xenorhabdus* and *Photorhabdus*.^21−27^ They are composed of nonpolar amino acids, l-valine (V), l-leucine (L), and l-phenylalanine (F) with often *N*-methylation (m) and C-terminal amines, phenylethylamine (PEA), tryptamine (TRA), tyramine (TYA), or agmatine (AGM).^21,23−25^ They exhibit high bioactivity against insect cells and malaria parasite, especially for the fully methylated RXPs.^21,23,25^ RXPs are assembled by NRPS systems composed of two to three single-module NRPS subunits.^21^ Each module consists of a starter C domain, an adenylation (A) domain often embedded with a methyltransferase (MT) domain, and a thiolation (T) domain, while the terminal module contains either an additional terminal C domain or is a freestanding C domain.^21^ We have reported recently that a very simple RXP-NRPS system from *Xenorhabdus* KJ12.1 can generate a variety of RXPs with different lengths and methylation patterns, suggesting the high flexibility of RXP-NRPS in this strain.^21^ Previous and latest NRPS engineering and structural studies have revealed that the ordered collinearity assembly line is directed by a specific pair of communication or docking domain (DD)s located at C- and N-terminus (^C^DD and ^N^DD) between two adjacent NRPS modules.^28−35^ Structural characterization of a DD pair between RXP-NRPSs from the KJ12.1 strain has illustrated that the flexibility of RXP production is a result of the promiscuous DD interactions between different RXP-NRPS subunits.^28^ Engineering of RXP-NRPSs via rational design based on these DD interactions was introduced as a new approach to make defined peptides via the exchange of flexible DD pairs against those with specific interactions among different NRPS modules.^29^

However, our attempts to further characterize RXPs in various *Xenorhabdus* and *Photorhabdus* strains revealed additional RXP-NRPS types. Some of them are highly flexible in the possible interaction with other NRPS subunits, resulting in diverse RXPs with different chain lengths and amino acid composition, while others are rather specific and produce only a small subset of RXPs. As it has been shown in our previous work for the flexible RXP-NRPS systems that the A-MT domains between different RXP-NRPSs can be exchanged against each other to produce new peptides with non-natural chemical diversity,^21^ our aim in this study was to use the specific RXP-NRPS system to generate a defined new RXP with different amino acids via the exchange of A-MT domains as a proof of concept. Therefore, we first chose the specific three single-module NRPS subunit system VietABC (GenBank accession code KT002577) from *X. vietnamensis* DSM 22392 reported previously to make a unique three amino acid-RXP, followed by peptide production analysis using HPLC-MS and structure confirmation by chemical synthesis. Additionally, we describe here the unique four single-module NRPS subunit system from *X. cabanillasii* DSM 17905, named CabABCD. We not only could express *cabABCD* (GenBank accession code KR871224) heterologously in *E. coli* and detect the RXPs as produced in the wild-type strain (WT) but also were able to obtain a unique four amino acid-RXP with four different amino acid residues after swapping all natural A-MT domains for those with different A domain specificities. The results provide a means of generating a defined RXP instead of an RXP library using a specific RXP-NRPS system and swapping of the A-MT domains. Additionally, the RXPs with amino acid residues in an unexpected order derived from the modified CabABCD system revealed an example of module skipping during the RXP biosynthesis.

## Results and Discussion

### Heterologous Expression of VietABC and Modified VietABC System

**Figure 1:**
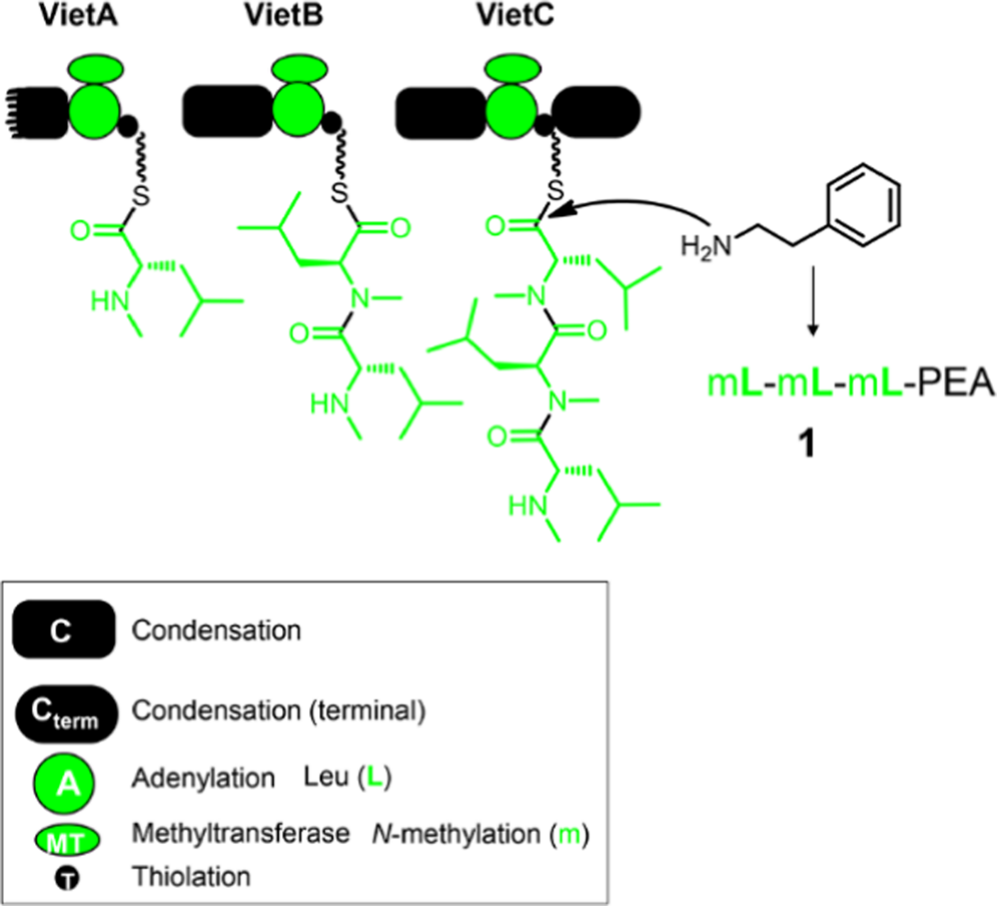
Proposed biosynthetic pathway of RXP **1** produced by the VietABC system.

**Figure 2:**
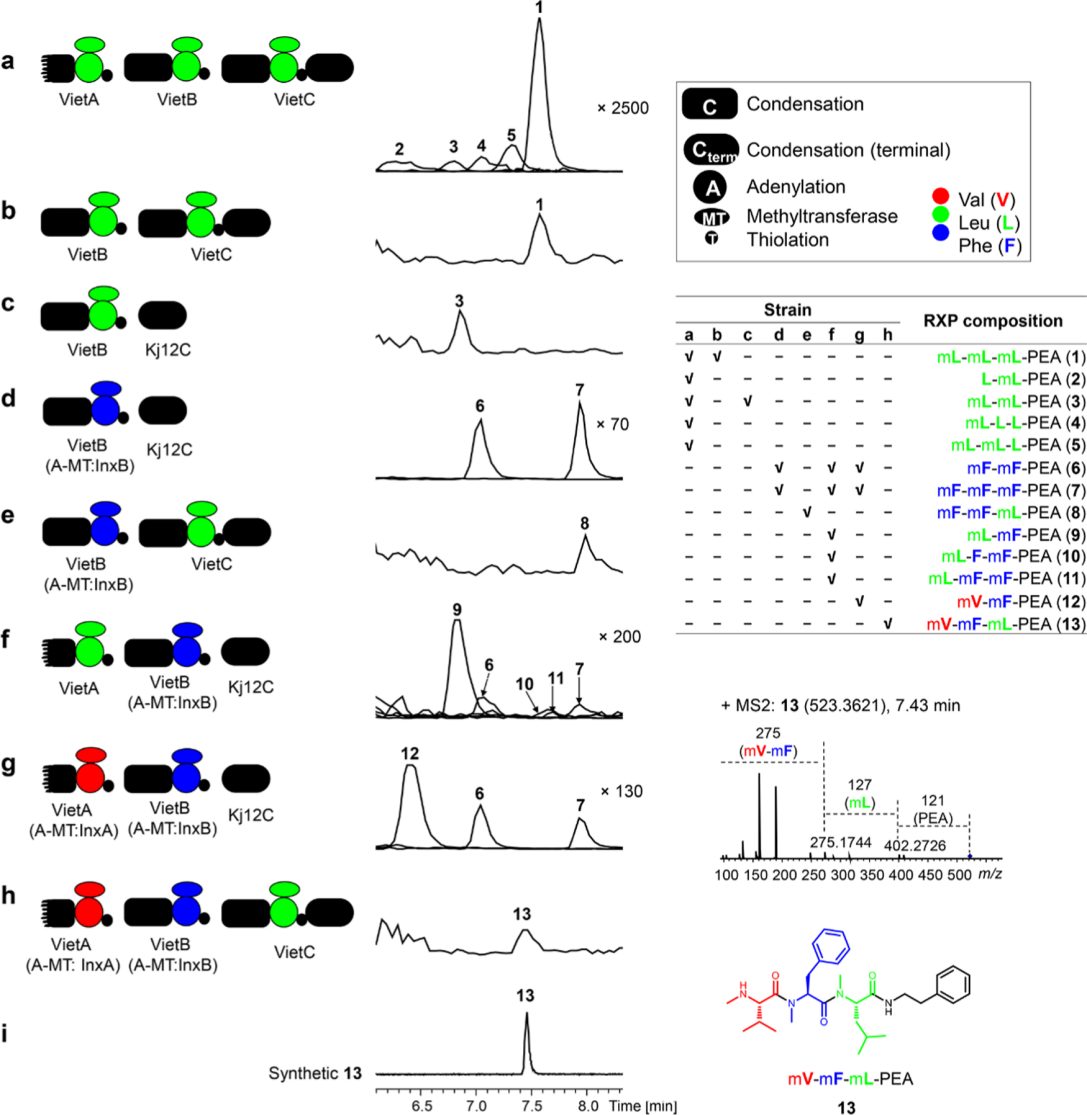
Generation of the tripeptide mV-mF-mL-PEA (**13**) using the VietABC system via the exchange of A-MT domain specificities. The genes encoding VietA, VietA (A-MT:InxA), VietB, VietB (A-MT:InxB), VietC, and Kj12C were separately cloned into different compatible expression vectors. (a–g) Coexpression of different NRPSs together for the production of RXPs to verify their specificities. (h) Coexpression of VietA (A-MT:InxA), VietB (A-MT:InxB), and VietC. (i) Synthetic RXP **13**. RXP-NRPS enzymology (left) and the produced RXPs as shown in HPLC-MS chromatograms (middle) and in tabulated form (right middle). The ×*n* means an *n*-fold increase in intensity, the chemical structure of **13**, and its MS/MS data were shown in the lower right corner and the domain architecture is explained in the upper right corner (condensation, C; adenylation, A; methyltransferase, MT; thiolation, T; and terminal condensation, Cterm domain), and the color code refers to the amino acid specificities of the A domains (Val, red; Leu, green; Phe, blue) that is also reflected by the peptides produced (right middle).

As reported before, VietABC is one of the relatively specific RXP systems.^3^ The biosynthetic pathways of selected RXPs **1** and **2** (Figures 1 and 2a,b) could also be deduced based on the model of N-/C-terminal docking domain (^C^DD/^N^DD) interactions described previously (Figure S1).^21,28^ In this system, truncated VietA is occasionally left out, VietB can act more than once, and MT domains embedded in VietB and VietC can be left out to generate five short Leu-containing RXPs **1**–**5** in total, of which the three methyl-Leu-containing peptide **1** is by far the major derivative (Figures 1 and 2a,b). Thus, VietABC was chosen to generate defined peptides. We observed that MT domains in RXP-NRPS systems including VietABC are all inserted between A8 and A9 core motifs of the A domains as described before (Figure S2).^36,37^ Therefore, we decided to make desired peptides consisting of different amino acids via A-MT di-domain exchange. A-MT domains in VietA and VietB were replaced by those in InxA and InxB from *X. innexi* DSM16336 specific for Val and Phe to get two new combinatorial biosynthetic systems, pCX107 [VietA (A-MT:InxA)] and pCX109 [VietB (A-MT:InxB)], respectively. As a test, VietA (A-MT:InxA) and VietB (A-MT:InxB) are validated with different systems, showing that VietB and its variant VietB (A-MT:InxB) can act two or three times when coexpressed together with VietC or Kj12C, leading to the production of three new RXPs **6**–**8** (Figure 2d,e). Unexpectedly, the expression of VietA and its variant VietA (A-MT:InxA) (Val specificity) separately in VietB(A-MT:InxB)-VietC and VietB(A-MT:InxB)-Kj12C systems resulted in the production of more RXPs (**6**–**12**) Figure 2f,g. However, when VietA (A-MT:InxA), VietB (A-MT:InxB), and VietC were coexpressed together, only a single RXP **13** was detected (Figure 2h). UPLC-MS/MS analysis of all compounds produced above showed that they are all RXPs as described previously with different *N*-methylation patterns, except for **13**. We analyzed the MS/MS fragmentation and elucidated the chemical structures for **6**–**13**. RXP **13** is a new fully methylated tripeptide mV-mF-mL-PEA (**13**) with an exact mass of 523.3640 [M + H]^+^ and the chemical formula of C_31_H_47_N_4_O_3_ (Figure 2h and Table S1). Further structural conformation of **13** was carried out by comparing the retention time between the product produced in *E. coli* and the chemically synthesized **13** (Figure 2h,i), which also allowed the quantification of the *E. coli* production to be about 0.1 μg/L.

By correlating the amino acid compositions of RXP **1**–**13** with the RXP-NRPS enzymology shown in Figure 2, we deduced that VietB and its variant VietB (A-MT:InxB) can act twice in most cases for the production of RXPs **1**, **3**, **6**, **8**, **10**, and **11** (Figures 2b–e and S3) sometimes even three times when VietB (A-MT:InxB) was coexpressed with a terminal Kj12C containing only one stand-alone C domain for the production of RXP **7** as shown in Figure 2d,f,g. These results indicated that VietB worked iteratively up to three times and then interacts with downstream VietC or Kj12C to obtain RXPs detected above. To check the relevance between DD interaction and iterative use of VietB, we generated DD interaction models based on our previous study^28,29^ for VietB-^C^DD/VietB-^N^DD, VietB-^C^DD/VietC-^N^DD, and VietB-^C^DD/Kj12C-^N^DD (Figure S1). The model showed that the key residues Q24 and E28 on the β2 sheet of VietB-^N^DD do not form any salt bridge with their interactive residues D and E on the β3 sheet of VietB-^C^DD, while the residue K28 or R24 from the β2 sheet of VietC-^N^DD or Kj12C-^N^DD can form at least one salt bridge with the corresponding residue E or D on the β3 sheet of VietB-^C^DD (Figure S1). Therefore, the salt bridges formed by key residues between ^C^DDs and ^N^DDs might not be the only major factors to mediate NRPS interactions for the production of RXPs but other factors might play a role as well. Nevertheless, the specific RXP-NRPS system could indeed be employed for the generation of RXP with amino acid diversity in defined length and order via the exchange of A-MT domains with different specificities. However, characterization of the overall structure of RXP-NRPS and interaction details between different domains and modules will be helpful in the future for an even better rational design of these NRPS systems to generate desired peptides.

### Heterologous Expression of CabABCD in *E. coli*

**Figure 3:**
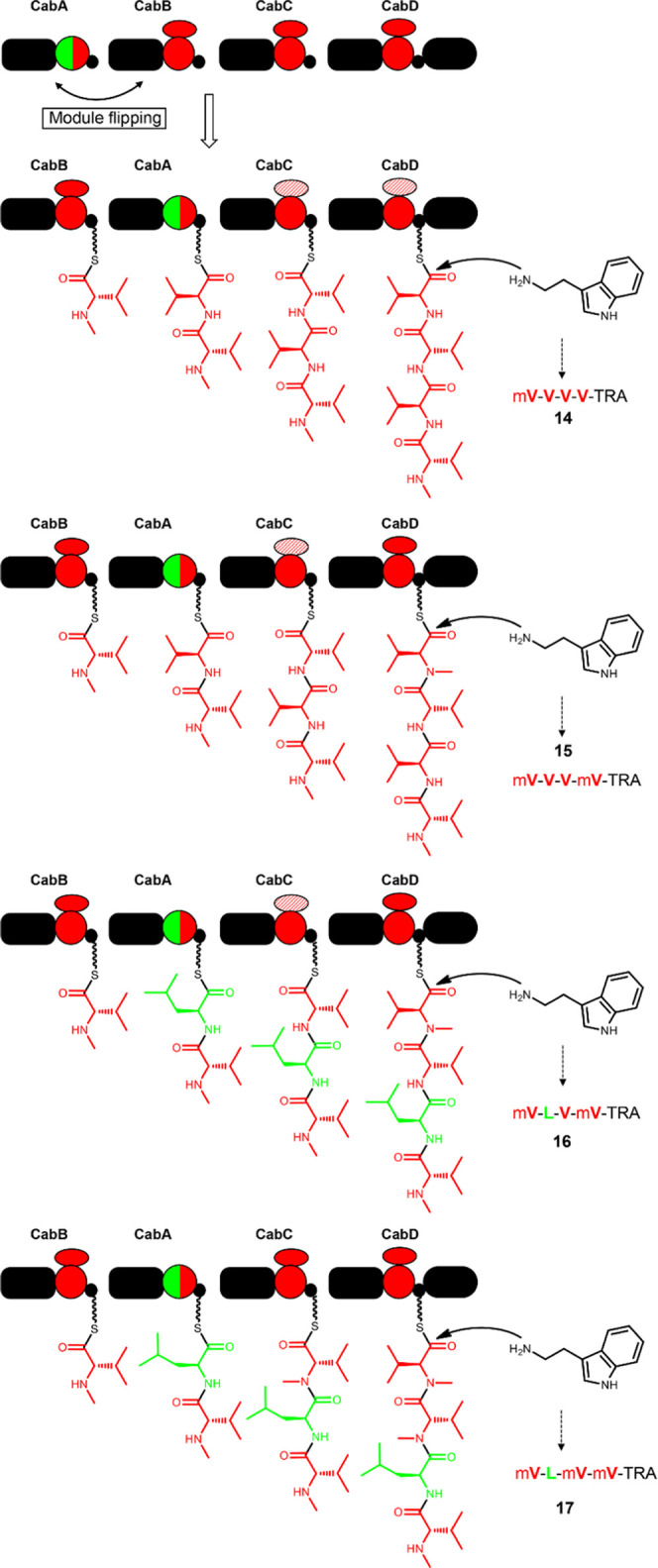
Proposed biosynthetic pathway of RXPs **14**–**17** produced in *X. cabanillasii* DSM 17905. Domains not used in the respective steps are shaded. A domain in CabA in half red and half green indicates the specificity for either Val or Leu.

**Figure 4:**
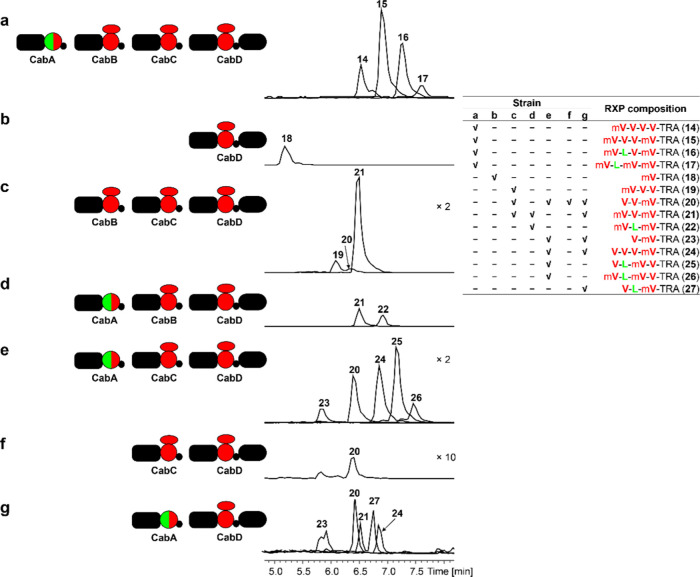
Heterologous expression of CabABCD in *E. coli* DH10B MtaA. (a) Expression of CabABCD. (b) Expression of CabD. (c) Coexpression of CabBC and CabD. (d) Coexpression of CabAB and CabD. (e) Coexpression CabA and CabCD. (f) Coexpression of CabC and CabD. (g) Coexpression of CabA and CabD. RXP-NRPS enzymology (left). The produced RXPs as shown in HPLC-MS chromatograms (middle) and in tabulated form (right top). The ×*n* indicates an *n*-fold increase in intensity, the domain architecture, and the color codes are the same as explained in Figure 2.

During our screening of RXP gene clusters in genome sequences of *Xenorhabdus* and *Photorhabdus* strains, we have identified a unique four single-module RXP-NRPSs in *X. cabanillasii* DSM 17905, named CabABCD. Previously, this gene cluster was described to consist of three genes as *cabABC* because of its incomplete and repetitive sequence.^21^ In the WT strain, only four RXPs **14**–**17** were detected (Figure S5), all of which contain four amino acid residues corresponding to the four single-module NRPSs. However, according to the general NRPS collinearity rule,^1,4^ the RXPs biosynthesized by CabABCD were supposed to be L-mV-mV-mV-TRA and V-mV-mV-mV-TRA. Since the structures of all RXPs were chemically confirmed by HPLC-MS and NMR analysis,^4^ it was speculated that the position of CabA and CabB are inverted during the biosynthesis of **14**–**17** (Figure 3). We also analyzed all DDs in CabABCD based on the known DD structure in RXP-NRPS from strain KJ12.1^7^ and predicted all possible DD interactions in this system, which can partially explain the promiscuity of RXPs derived from CabABCD based on strong or weak interaction using salt bridges (Figure S4). To further investigate the biosynthesis of RXPs in the CabABCD system, heterologous expression of *cabABCD* in *E. coli* was performed. As the *cab* gene cluster is relatively large for direct cloning, we split the entire gene cluster into two parts *cabAB* and *cabCD* and assembled them under the control of arabinose inducible promoter (*P*_*BAD*_) on two separate and compatible vectors and introduced both plasmids into *E. coli* DH10B MtaA. The LC-MS analysis of the resulting culture extracts resulted in almost the same production of RXPs as in the WT (Figures 4a and S5).

### Production of a New and Specific RXP Using CabABCD in *E. coli*

**Figure 5:**
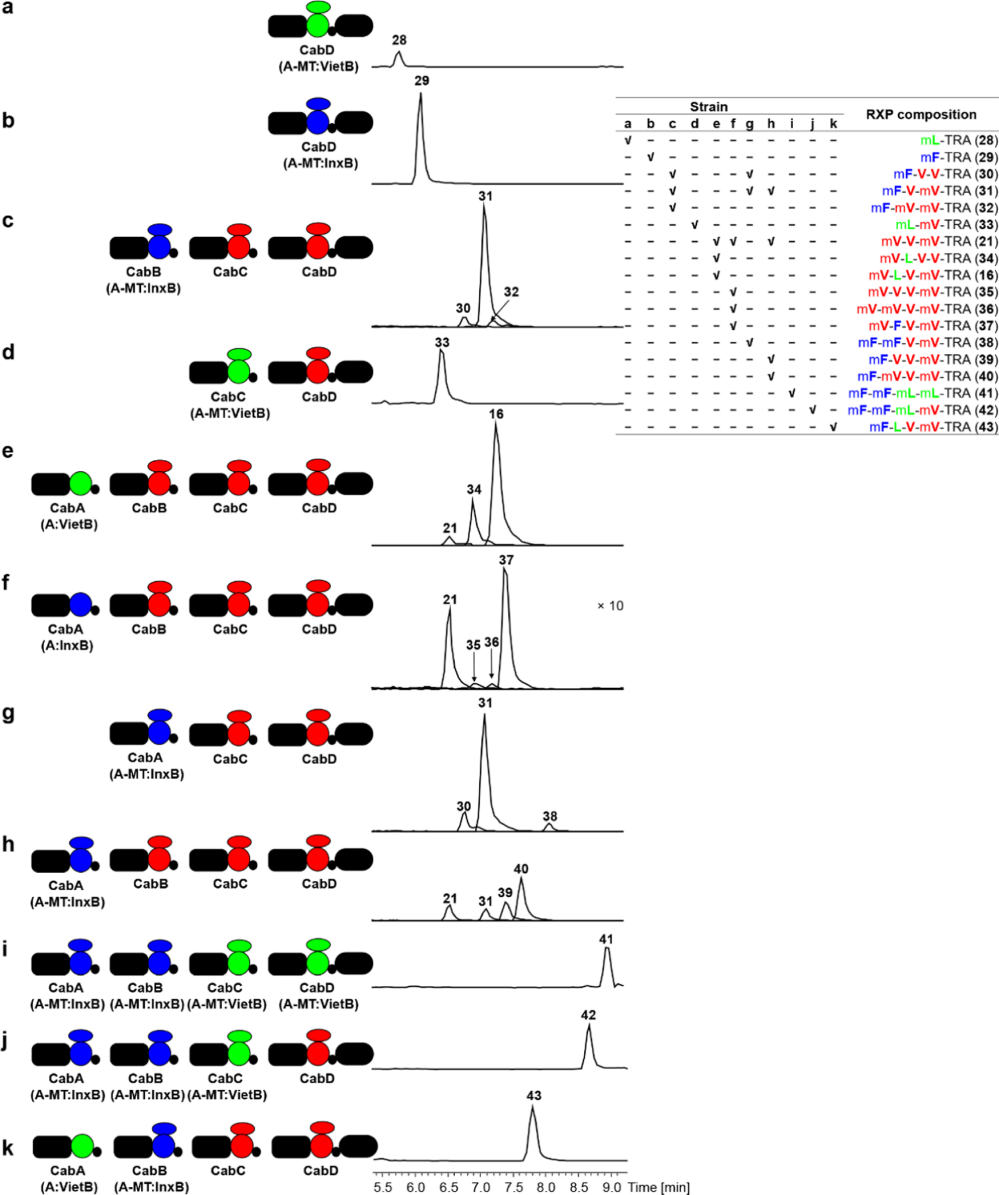
Heterologous expression of modified CabABCD in *E. coli* DH10B MtaA. (a) Expression of CabD with A-MT domain replaced by that from VietB. (b) Expression of CabD with A-MT domain replaced by that from InxB. (c) Coexpression of CabB (A-MT:InxB) and CabCD. (d) Coexpression of CabC (A-MT:VietB) and CabD. (e) Coexpression of CabA (A:VietB), CabBC, and CabD. (f) Coexpression of CabA (A:InxB), CabBC, and CabD. (g) Coexpression of CabA (A-MT:InxB) and CabCD. (h) Coexpression of CabA (A-MT:InxB), CabBC, and CabD. (i) Coexpression of fully modified CabABCD, CabA (A-MT:InxB), CabB (A-MT:InxB), CabC (A-MT:VietB), and CabD (A-MT:VietB). (j) Coexpression of CabA (A-MT:InxB), CabB (A-MT:InxB), CabC (A-MT:VietB), and CabD. (k) Coexpression of CabA (A:VietB), CabB (A-MT:InxB), CabC, and CabD. RXP-NRPS enzymology (left) and the produced RXPs as shown in HPLC-MS chromatograms (middle) and in tabulated form (right top). The ×*n* indicates an *n*-fold increase in intensity, the domain architecture, and the color codes are the same as explained in Figure 2.

To make a new and specific RXP using CabABCD as described above for VietABC, we individually constructed four plasmids carrying CabA, CabB, CabC, and CabD with natural A or A-MT domains for Val or Leu specificity replaced by A or A-MT domains from VietB or InxB for Leu or Phe specificity to get CabA (A:VietB), CabA (A:InxB), CabA (A-MT:InxB), CabB (A-MT:InxB), CabC (A-MT:VietB), CabD (A-MT:VietB), and CabD (A-MT:InxB) (Figure 5 and Table S5). Meanwhile, the coding genes for natural CabA, CabB, CabC, CabD, CabBC, and CabCD were also cloned into different vectors (Figure 4) for further comparison of RXP production following combinatorial biosynthesis.

To verify the substrate specificity, *E. coli* strains carrying *cabD* were cultivated for the production of short RXP with the addition of 0.1% arabinose and 1 mM TRA. As a result, one amino acid containing RXP, mV-TRA (**18**) was detected in the culture (Figure 4b). Then, we confirmed the functions of other *cabA*-, *cabB*-, and *cabC*-derived constructs via stepwise coexpression of them with *cabD* to detect the production of longer RXPs **19**–**27** (Figure 4c–g). The same procedures were done for the variants of CabD, CabA, CabB, and CabC to get RXPs **28**–**41** (Figure 5). It was shown that CabAB, CabBC, CabAC, CabC, and CabA as well as their variants could individually interact with CabD to make RXPs (Figures 4 and 5). In addition, we observed that natural CabA could act two or three times to generate RXPs **24**–**26** and RXPs **20**, **21**, **27**, and **24** in three or two subunit systems CabACD and CabAD (Figure 4e,g) as well as iterative use (twice) of CabC in CabCD system, but not in four modular CabABCD and A-MT domain exchanged variants (Figures 4a and 5c–i), which might reflect the relative flexibility of domain interactions in the natural system.

To investigate the exchange of positions between CabA and CabB, we first made an alignment of all A-MT domains with different specificities from selected RXP-NRPSs and defined the boundary between A and MT domains (Figure S2). The A domain in CabA was separately replaced by those from VietB and InxB for Leu and Phe specificities (Figure 5e,f). The resulting strains showed the production of four amino acid-containing RXPs **16**, **34**, and **37** with Leu or Phe at the second position of RXPs, confirming the order of NRPS subunits as CabBACD. However, when the A domain in CabA was exchanged against the full A-MT domain from InxB the two RXPs **39** and **40** were observed (Figure 5h), indicating the order of these NRPSs as CabABCD. This suggests that similar to the modified VietABC system, the addition of an MT domain in CabA might be involved in protein–protein interaction in addition to the DDs.^38,39^ With respect to the DD interaction, CabA can either be first or second in the biosynthesis with strong interactions with CabB (Figure S4). However, the mechanism behind this hypothesis needs to be further investigated via the structural characterization of the interaction between two adjacent RXP-NRPSs.

Finally, to make a completely new specific RXP relative to those produced in the WT, we expressed modified CabABCD, CabA (A-MT:InxB), CabB (A-MT:InxB), CabC (A-MT:VietB), and CabD (A-MT:VietB) and detected a new fully methylated Leu and Phe containing RXP **41** (Figure 5i). Further combination of modified CabABCD systems led to the production of new specific RXPs **42** and **43** (Figure 5j,k). All RXPs were identified by labeling experiments and MS/MS fragmentation as described previously (Figures S6 and S7).^21,22^ The structures of selected RXPs **16**, **22**, **31**, and **41** were further confirmed by chemical synthesis (Figure S8), and their production levels in *E. coli* were quantified as ∼99.75 mg/L (**16**), 5.69 mg/L (**22**), 29.72 mg/L (**31**), and 4.70 mg/L (**41**), respectively.

### Functional Analysis of Methyltransferase Domains in CabABCD

**Figure 6:**
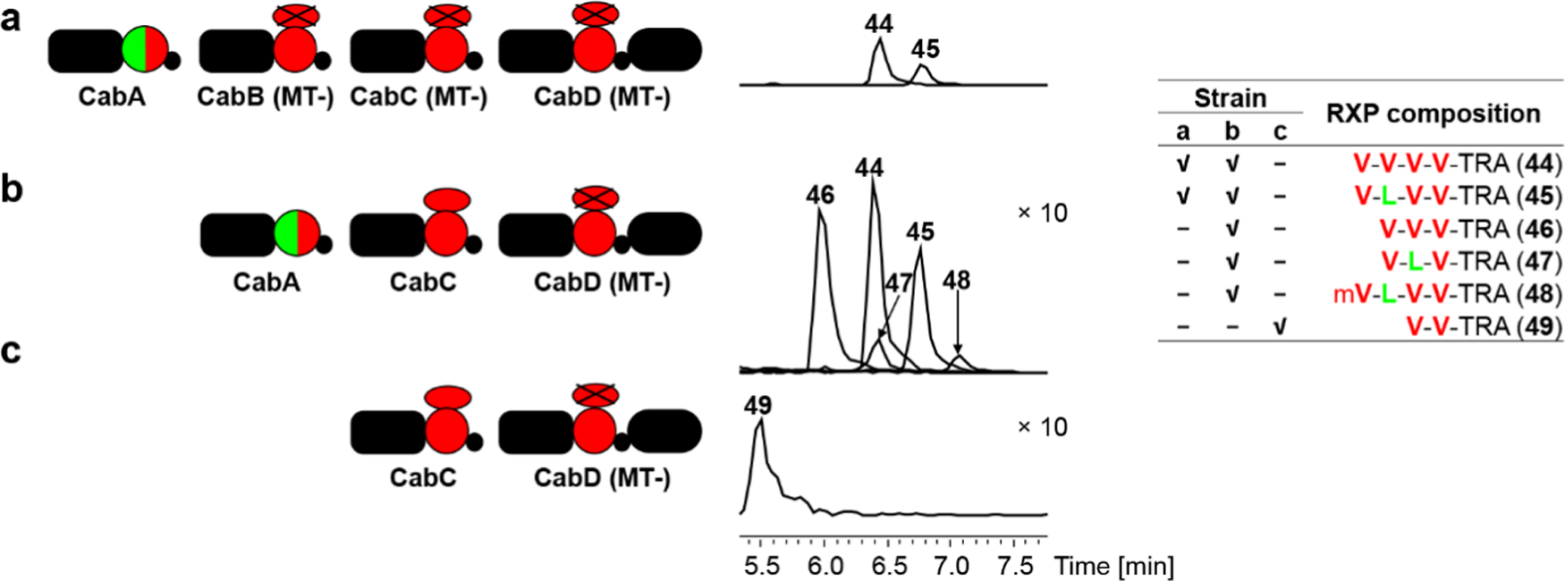
Heterologous expression of MT-deficient CabABCD in *E. coli* DH10B MtaA. (a) Coexpression of CabA, CabB (MT-), CabC (MT-), and CabD (MT-). (b) Coexpression of CabA, CabC, and CabD (MT-). (c) Coexpression of CabC and CabD (MT-). RXP-NRPS enzymology (left) and the produced RXPs as shown in HPLC-MS chromatograms (middle) and in tabulated form (right top). The ×*n* indicates an *n*-fold increase in intensity. The domain architecture and the color codes are the same as explained in Figure 2.

Of note, during the analysis of RXPs produced by the CabABCD system, we observed that RXPs with Val incorporated by CabC were barely methylated but Val incorporated by CabB and CabD were *N*-methylated in most cases. To look into the methylation patterns of RXPs and investigate the functional abilities of all MT domains in CabBCD, we made constructs with point mutations resulting in the modification of the conserved motifs of LLEIGCGSGLL (CabB and CabD) or LLEIGCGTGVL (CabC) with all three G replaced by three S in all MT domains to get CabB (MT-), CabC (MT-), and CabD (MT-). Coexpression of CabA, CabB (MT-), CabC (MT-), and CabD (MT-) resulted in the production of RXPs **44** and **45** without any methylation as expected (Figure 6a), which demonstrated the essential role of MT domains in the methylation in RXP biosynthesis. To check the efficacy of CabC-MT, we first coexpressed CabA, CabC, and MT-deficient CabD, resulting in the detection of Val/Leu-containing RXPs **44**–**47** without any methylation and a minor derivative **48** containing one methylated Val (Figure 6b). In this system, we noticed that RXPs **44**–**45** and **48** all contain four amino acids, which probably resulted from iterative use (twice) of CabA as observed in Figure 4e. Further coexpression of CabC alone with deficient CabD (MT-) led to the production of unmethylated **49** (Figure 6c). The results suggested the weak activity of the CabC-MT domain.

## Conclusions

Taken together, the present study not only reports another type of RXP-NRPS system with specific interaction relative to the highly flexible systems described before but also a unique four modular RXP-NRPS system identified in *X. cabanillasii*. Additionally, we can use these specific systems as platforms to produce new RXPs with defined lengths and amino acid specificities via swapping of A or A-MT domain specificities. Moreover, our results showed that in the CabABCD system, the order of NRPS subunits can be changed probably dependent on the CabA A domain, suggesting a new mechanism of the NRPS protein–protein interaction. Recent structural characterization of truncated intermodule NRPS system suggested that the conformational flexibility within the two adjacent modules may allow possible transient interactions between non-neighboring NRPS modules, leading to the production of NRPs in a nonlinear fashion.^40,41^ Thus, to get deeper insights into the detailed RXP biosynthetic mechanisms, further combined experiments including crystallography and cryoelectron microscopy is required to characterize the structures of individual RXP-NRPS module and dimodular complex. With this information in hand, one can properly design and engineer the RXP-NRPS system in combination with classic NRPSs to make any desired bioactive peptides.

## Methods

### General Molecular Biology

Cultivation of *Xenorhabdus* and *E. coli* strains (Table S3) was carried out as described previously.^21^ Procedures, such as plasmid DNA preparation, transformation, restriction digestion, and DNA gel electrophoresis were adapted from standard protocols.^42^ Isolation of genomic DNA was carried out according to the manufacturer’s instructions (QIAGEN). Phusion high-fidelity DNA polymerases (Thermo Scientific) were used for PCR amplifications. PCR primers used in this study are listed in Table S4. All of the plasmids (Table S5) generated in this study were constructed via Gibson assembly.^43^ The basic cloning was performed in *E. coli* DH10B MtaA strain.^21,22,29,44^

### Construction of the Heterologous Expression Systems of *vietABC* and *cabABCD*

Genes, *vietA*, *vietB*, and *vietC* were separately cloned into the vectors, pCOLA-ara-tacI, pACYC-ara-tacI, and pCDF-ara-tacI under the control of *pBAD* promoter to generate plasmids of pCX106, pCX107, and pCX99, respectively (Table S5). For the expression of *cabABCD*, different plasmids were constructed by introductions of *cabAB*, *cabCD*, *cabBC*, and *cabD* into pCOLA-ara-tacI, pCDF-ara-tacI, pACYC-ara-tacI, and pCDF-ara-tacI to get pCXLZ1, pCXLZ3, pCXLZ2, and pCXLZ4 (Table S5), respectively.

### A-MT Domain Swaps in VietABC and CabABCD for the Generation of Specific New RXPs

Plasmids pCX107 and pCX109 (Table S5) were constructed by the exchange of DNA fragments for A-MT domains with leucine specificities from VietA and VietB against those from InxA and InxB activating Val and Phe, respectively. Plasmids pCXLZ5–14 (Table S5) were created by the replacement of DNA fragments for A-MT domains with Val specificities from CabABCD by those from VietB and InxB activating Leu and Phe, respectively. *E. coli* DH10B MtaA was co-transformed with plasmids with different combinations for the production of RXPs.

### MT Domain Mutation in the CabABCD System

Point mutations on MT of CabBCD were carried out by the replacement of three Gly in the conserved motif of LLEIGCGS(T)GLL(V)L with three Ser as described previously.^21^

### Heterologous Production of RXPs

Heterologous production of RXPs was conducted by the inoculation of overnight culture (1:100) from *E. coli* DH10B MtaA strain carrying specific plasmids into 10 mL of fresh LB medium supplemented with appropriate antibiotics, 1 mM phenylethylamine (PEA) or 1 mM tryptamine (TRA), 2% (v/v) of Amberlite XAD-16 resin (Sigma-Aldrich), 0.1% of l-arabinose for inducing the expression of RXP-NRPSs, and followed by growing the culture at 30 °C, 1 d, and 200 rpm for the production of RXPs. For isotopic labeling of RXPs, the culture was additionally fed with l-methionine-(*methyl*-d_3_) (Isotec), l-leucine-d_10_ (Sigma-Aldrich), l-valine-d_8_ (Sigma-Aldrich), or l-phenylalanine-d_8_ (Sigma-Aldrich).^21,24^

### Culture Extraction and HPLC-MS Analysis

The bacterial cell pellets and XAD beads were collected after centrifugation and resuspended in 10 mL of methanol. XAD beads were washed with methanol by inverting for 1 h, followed by separating from methanol through filter paper. The resulting methanol extracts were evaporated to dryness and redissolved in 1 mL of fresh methanol. The methanol extracts obtained above (1 mL) were cleaned up via centrifugation at 17 000*g* for 20 min. Twenty microliters of crude extracts was diluted in 180 μL of methonal before analysis, 5 μL of which was injected and analyzed by HPLC-ESI-MS by a Dionex UltiMate 3000 system coupled to a Bruker AmaZon X mass spectrometer or HPLC-ESI-HRMS (Impact II) using an ACQUITY UPLC BEH C18 column (130 Å, 2.1 mm × 100 mm, 1.7 μm particle size, Waters GmbH) at a flow rate of 0.6 mL/min using acetonitrile and water containing 0.1% formic acid (v/v) in a gradient ranging from 5 to 95% of acetonitrile (ACN) over 16 min. Spectra for RXPs were recorded in a positive ion mode with the range from 100 to 1200 *m/z* and UV at 200–600 nm.

### Chemical Synthesis of Short RXPs

The synthesis was performed manually by employing standard Fmoc solid-phase peptide synthesis (SPPS) as described previously.^45^ For a schematic overview, see Figure S9. Briefly, step **a** is the attachment of the C-terminal amine PEA on the DFPE resin. Step **b** is the acylation of the first amino acid with C-terminal amine PEA. Step **c** is the coupling of amino acids to the peptide sequence. The final step **d** is the cleavage of the peptide from the resin. The resin was removed by filtration and the solution was concentrated in vacuo. The residue was purified by a semipreparative Agilent HPLC system. The structures of pure compounds were confirmed by HRMS and H^1^ and C^13^ NMR.

### Quantification of Production of RXPs

Compounds **13**, **16**, **22**, **31**, and **41** are RXPs produced in *E. coli*. To calculate their absolute production titres in *E. coli*, the pure synthetic compounds **13**, **22**, **31**, **41**, and isolated **16** from *X. cabanillasii*(6) were prepared at different concentrations (100, 50, 25, 12.5, 6.25, 3.125, 1.56, 0.78, 0.39, and 0.039 μg/mL) and measured by HPLC-MS. The peak area for each compound at different concentrations was calculated using Bruker Compass Data Analysis 4.0 program to generate the equations for compounds **13**, **16**, **22**, **31**, and **41** with *y* = 9*E* + 08*x* (*R*^2^ = 0.9849), *y* = 1*E* + 09*x* (*R*^2^ = 0.9801), *y* = 1*E* + 09*x* (*R*^2^ = 0.9884), *y* = 1*E* + 09*x* (*R*^2^ = 0.9788), and *y* = 1*E* + 09*x* (*R*^2^ = 0.9840), respectively. The samples of crude extract from each strain were prepared as described above and analyzed by HPLC-MS. The peak area of the expected compound was obtained and its corresponding production titer was calculated based on the equation generated from the standard compounds with **13**, **16**, **22**, **31**, and **41**.

## Supporting Information Available

Characterization of synthetic RXPs **13**, **22**, **31**, and **41**; HRMS data and chemical formula of all RXPs; relative production of RXPs in different strains; bacterial strains, primers, and plasmids used; DD interactions in VietABC and CabABCD; sequence alignment of A and A-MT domains from selected classic NRPSs and RXP-NRPSs; HPLC-MS/MS analysis; isotope labeling experiments; chemical synthesis routes; and ^1^H and ^13^C NMR spectra of synthetic RXPs **13**, **22**, **31**, and **41** (PDF)
