# Supplementary material for: Engineering of Specific Single-Module Nonribosomal Peptide Synthetases of the RXP Type for the Production of Defined Peptides
Source: ACS Synth Biol. 2022 Dec 19;12(1):203–12. doi: 10.1021/acssynbio.2c00472 (PMC9872161; doi:10.1021/acssynbio.2c00472)
Supplement: Supplementary file 1 — sb2c00472_si_001.pdf [file sb2c00472_si_001.pdf]

Supporting Information

Engineering of Specific Single-Module Nonribosomal Peptide Synthetases of the RXP-type for the Production of Defined Peptides

Xiaofeng Cai,[†,‡,#,*] Lei Zhao,[‡,§,#] and Helge B. Bode[‡,∥,⊥,^,*]

Affiliations:

[†]School of Pharmacy, Tongji Medical College, Huazhong University of Science and Technology, 430030 Wuhan, China

[‡]Molecular Biotechnology, Department of Biosciences, Goethe University Frankfurt, 60438 Frankfurt am Main, Germany

[§]State Key Laboratory of Bio-organic and Natural Products Chemistry, Shanghai Institute of Organic Chemistry, Chinese Academy of Sciences, 200032 Shanghai, China

[∥]Department of Natural Products in Organismic Interactions, Max-Planck-Institute for Terrestrial Microbiology, 35043 Marburg, Germany

[⊥]Chemical Biology, Department of Chemistry, Philipps University Marburg, 35037 Marburg, Germany

^Senckenberg Gesellschaft für Naturforschung, 60325 Frankfurt, Germany

[#]These authors contributed equally to this work

Correspondence to:

Helge B. Bode, Email: helge.bode@mpi-marburg.mpg.de

Xiaofeng Cai, Email: caixiaofeng@hust.edu.cn

Table of Contents

Supplementray Methods **3**

Characterization of synthetic RXPs. 3

Supplementray Tables **6**

Table S1. HR-Masses and chemical formula of compounds in this study. 6

Table S2. Relative production of RXPs in different strains, related to Figure 1. 8

Table S3. Bacterial strains used in this study. 9

Table S4. Primers used in this study. 10

Table S5. Plasmids used in this study. 16

Supplementary Figures **19**

Figure S1. Docking domain (DD) interactions in VietABC system and VietB-^{C}DD/Kj12C-^{N}DD 19

Figure S2. Sequence alignment of A and A-MT domains from selected classic NRPSs and RXP-NRPSs. 20

Figure S3. MS/MS analysis of RXPs **1**, **3**, **6**, **8**, **10**–**11** and **7**.. 20

Figure S4. Docking domain (DD) interactions in CabABCD 22

Figure S5. HPLC-MS/MS analysis of RXPs **14**–**17** 23

Figure S6. Selected examples of MS data from isotopic labeling experiments 24

Figure S7. Structural elucidation of selected RXPs based on MS/MS fragmentations 25

Figure S8. Final structural confirmation of major RXPs in selected constructs 27

Figure S9. Synthesis of model mV-mF-mL-PEA (**13**) 28

Figure S10. ^{1}H NMR spectrum of compound **13** (MeOD, 400 MHz) 29

Figure S11. ^{13}C NMR spectrum of compound **13** (MeOD, 100 MHz). 29

Figure S12. ^{1}H NMR spectrum of compound **22** (MeOD, 500 MHz) 30

Figure S13. ^{13}C NMR spectrum of compound **22** (MeOD, 125 MHz). 30

Figure S14 ^{1}H NMR spectrum of compound **31** (MeOD, 500 MHz) 31

Figure S15. ^{13}C NMR spectrum of compound **31** (MeOD, 125 MHz). 31

Figure S16. ^{1}H NMR spectrum of compound **41** (MeOD, 500 MHz) 32

Figure S17. ^{13}C NMR spectrum of compound **41** (MeOD, 125 MHz). 32

References **33**

Supplementary Methods

Characterization of Synthetic RXPs 13, 22, 31 and 41

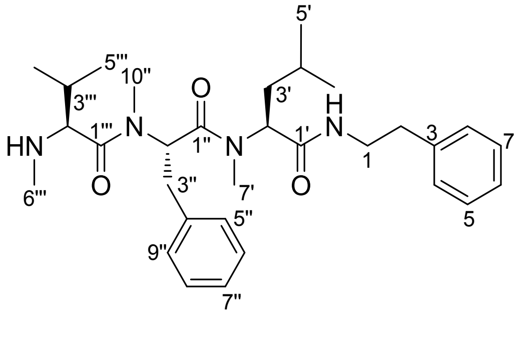

mV-mF-mL-PEA (**13**)

Compound **13** was obtained as a white solid; ^{1}H NMR (MeOD, 400 MHz) δ 7.35-7.18 (10H; H-4, 5, 6, 7, 8, 5", 6", 7", 8", 9"), 5.99 (dd, *J* = 9.5, 6.3 Hz, 1H; H-2"), 5.13 (dd, *J* = 10.0, 5.9 Hz, 1H; H-2'), 4.09 (d, *J* = 4.1 Hz, 1H; H-2'''), 3.46 (m, 2H; H-1), 3.13 (m, 2H; H-3"), 3.06 (s, 3H; H=10"), 2.86 (s, 3H; H-7'), 2.82 (m, 2H; H-2), 2.08 (m, 1H; H-3'''), 1.93 (s, 3H; H-6'''), 1.63 (m, 2H; H-3'), 1.28 (m, 1H; H-4'), 1.09 (d, *J* = 7.0 Hz, 3H; H-4'''), 0.98 (d, *J* = 7.0 Hz, 3H; H-5'''), 0.93 (d, *J* = 6.7 Hz, 3H; H-5'), 0.90 (d, *J* = 6.5 Hz, 3H; H-6'); ^{13}C NMR (MeOD, 100 MHz) δ 172.7 (C1'), 172.4 (C1"), 168.6 (C1'''), 140.6 (C3), 138.3 (C4"), 130.9 (C5", C9"), 130.1 (C4, C8), 129.8 (C6", C8"), 129.7 (C5, C7), 128.2 (C7"), 127.6 (C6), 65.2 (C2'''), 56.5 (C2'), 56.1 (C2"), 42.0 (C1), 38.3 (C3'), 36.5 (C2), 35.5 (C3"), 32.9 (C-6'''), 31.6 (C7'), 31.4 (C10"), 31.3 (C3'''), 26.3 (C4'), 23.7 (C5'), 22.0 (C6'), 19.3 (C4'''), 17.3 (C5'''); HRESIMS *m/z* 523.3641 [M + H]$^{+}$ (calcd for $C_{31}H_{47}N_4O_3$, 523.3643).

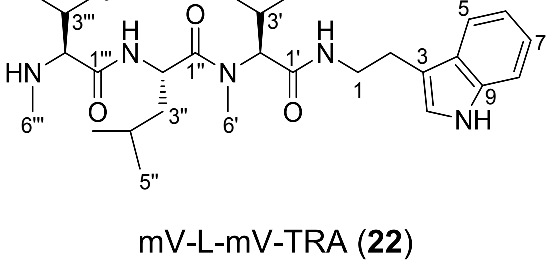

mV-L-mV-TRA (**22**)

Compound **22** was obtained as a yellow solid; ^{1}H NMR (MeOD, 500 MHz) δ 7.55 (d, *J* = 7.9 Hz, 1H; H-5), 7.32 (d, *J* = 8.1 Hz, 1H; H-8), 7.10-6.96 (3H; H-6, 7, 10), 4.90 (1H; H-2''), 4.54 (d, *J* = 11.1 Hz, 1H; H-2'), 3.50 (3H; H-1, 2'''), 3.14 (s, 3H; H-6'), 2.92 (t, *J* = 7.2 Hz, 2H; H-2), 2.59 (s, 3H; H-6'''), 2.23 (m, 1H; H-3'), 2.14 (m, 1H; H-3'''), 1.61 (m, 2H; H-3'', 4''), 1.36 (m, 1H; H-3''), 1.04 (d, *J* = 7.5 Hz, 3H; H-5'''), 0.99 (d, *J* = 6.9 Hz, 3H; H-4'''), 0.93 (6H; H-5'', 6''), 0.88 (d, *J* = 6.5 Hz, 3H; H-4'), 0.79 (d, *J* = 6.6 Hz, 3H; H-5'); ^{13}C NMR (MeOD, 125 MHz) δ 174.9 (C1''), 171.9 (C1', 1'''), 138.4 (C9), 128.9 (C4), 123.4 (C10), 122.5 (C7), 119.8 (C6), 119.4 (C5), 113.1 (C3), 112.4 (C8), 68.7 (C2'''), 64.2 (C2'), 49.7 (C2''), 41.3 (C3''), 41.0 (C1), 33.5 (C-6'''), 31.8 (C-3'''), 31.4 (C6'), 27.6 (C3'), 26.5 (C2), 26.2 (C4''), 23.7 (C5''), 21.9 (C6''), 19.9 (C4'), 19.3 (C5'), 18.8 (C5'''), 18.6 (C4'''); HRESIMS *m/z* 500.3591 $[M + H]^+$ (calcd for $C_{28}H_{46}N_5O_3$, 500.3595).

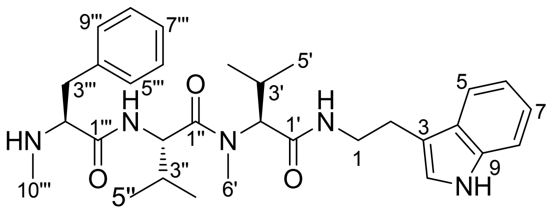

mF-V-mV-TRA (**31**)

Compound **31** was obtained as a yellow solid; ^{1}H NMR (MeOD, 500 MHz) δ 7.54 (d, *J* = 7.9 Hz, 1H; H-5), 7.34-7.20 (6H; H-5''', 6''', 7''', 8''', 9''', 8), 7.07 (2H; H-7, 10), 6.99 (t, *J* = 7.4 Hz, 1H; H-6), 4.64 (d, *J* = 7.7 Hz, 1H; H-2''), 4.59 (d, *J* = 11.1 Hz, 1H; H-2'), 3.98 (t, *J* = 6.6 Hz, 1H; H-2'''), 3.50 (m, 2H; H-1), 3.17 (1H; H-3'''), 3.14 (s, 3H; H-6'), 3.00 (1H; H-3'''), 2.92 (t, *J* = 7.2 Hz, 2H; H-2), 2.54 (s, 3H; H-10'''), 2.24 (m, 1H; H-3'), 1.93 (m, 1H; H-3''), 0.92-0.78 (12H; H-4'', 5'', 4', 5'); ^{13}C NMR (MeOD, 125 MHz) δ 174.1 (C1''), 171.9 (C1'), 169.7 (C1'''), 138.4 (C9), 135.7-128.9 (C4''', 5''', 6''', 7''', 8''', 9''', 4), 123.4 (C10), 122.5 (C7), 119.7 (C6), 119.4 (C5), 113.0 (C3), 112.4 (C8), 64.3 (C2'''), 64.0 (C2'), 56.6 (C2''), 41.0 (C1), 38.4 (C-3'''), 33.4 (C-10'''), 32.1 (C3''), 31.6

(C6'), 27.6 (C3'), 26.5 (C2), 19.8-18.7 (C4'', 5'', 4', 5'); HRESIMS *m/z* 534.3435 [M + H]$^+$ (calcd for $C_{31}H_{44}N_5O_3$, 534.3439).

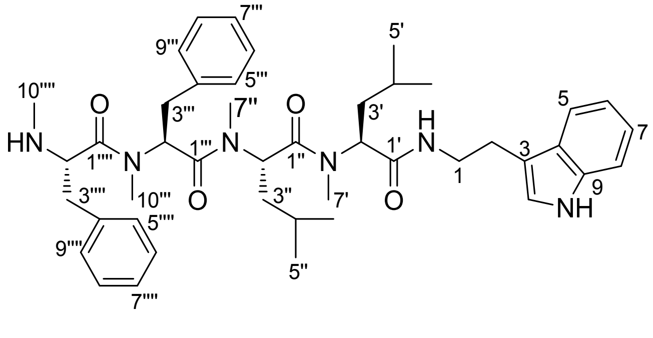

mF-mF-mL-mL-TRA (**41**)

Compound **41** was obtained as a yellow solid; ^{1}H NMR (MeOD, 500 MHz) δ 7.54 (d, *J* = 7.9 Hz, 1H; H-5), 7.40-7.14 (11H; H-5'''', 6'''', 7'''', 8'''', 9'''', 5''', 6''', 7''', 8''', 9''', 8), 7.11-6.92 (3H; H-6, 7, 10), 5.81 (t, *J* = 7.7 Hz, 1H; H-2'''), 5.36 (dd, *J* = 8.4, 6.2 Hz, 1H; H-2''), 4.98 (dd, *J* = 10.1, 5.9 Hz, 1H; H-2'), 4.63 (1H; H- H-2''''), 3.47 (m, 2H; H-1), 3.16 (3H; H-3'''', 3'''), 2.92 (3H; H-3''', 2), 2.86 (s, 3H; H-10'''), 2.68 (s, 3H; H-7''), 2.36 (s, 3H; H-7'), 2.29 (s, 3H; H-10''''), 1.59 (m, 1H; H-3''), 1.48 (m, 2H; H-3'), 1.38 (m, 1H; H-3''), 0.97-0.80 (12H; H-5'', 6'', 5', 6'); ^{13}C NMR (MeOD, 125 MHz) δ 173.0 (C1'', 1'), 170.1 (C1'''), 169.3 (C1''''), 138.3 (C9), 138.1 (C4'''), 134.8 (C4''''), 130.9-128.2 (C5'''', 6'''', 7'''', 8'''', 9'''', 5''', 6''', 7''', 8''', 9''', 4), 123.6 (C10), 122.5 (C7), 119.8 (C6), 119.5 (C5), 113.2 (C3), 112.4 (C8), 60.8 (C2''''), 56.6 (C2''', C2'), 53.6 (C2''), 41.5 (C1), 39.1 (C3''), 38.5 (C-3'), 37.8 (C3''''), 36.3 (C3'''), 32.4 (C10''''), 31.4 (C10''', C7'), 31.1 (C7''), 26.2 (C2), 26.0 (C4''), 23.9-21.8 (C5'', 6'', 5', 6'); HRESIMS *m/z* 737.4721 [M + H]$^+$ (calcd for $C_{44}H_{61}N_6O_4$, 737.4749).

Supplementary Tables

Table S1. HR-Masses and chemical formula of compounds in this study.

Compound	Calculated mass $[M+H]^+$	Detected mass $[M+H]^+$	Chemical formula	RDB	Δppm
**1**	503.3956	503.3947	$C_{29}H_{51}N_4O_3$	6.5	1.7
**2**	362.2794	362.2802	$C_{21}H_{36}N_3O_2$	5.5	2.3
**3**	376.2959	376.2952	$C_{22}H_{38}N_3O_2$	5.5	1.8
**4**	475.3643	475.3632	$C_{27}H_{47}N_4O_3$	6.5	2.3
**5**	489.3787	489.3799	$C_{28}H_{49}N_4O_3$	6.5	2.4
**6**	444.2646	444.2630	$C_{28}H_{34}N_3O_2$	13.5	3.4
**7**	605.3446	605.3457	$C_{33}H_{45}N_6O_5$	14.5	-1.8
**8**	571.3643	571.3621	$C_{35}H_{47}N_4O_3$	14.5	3.5
**9**	410.2802	410.2783	$C_{25}H_{36}N_3O_2$	9.5	4.6
**10**	557.3465	557.3486	$C_{34}H_{45}N_4O_3$	14.5	3.9
**11**	571.3643	571.3622	$C_{35}H_{47}N_4O_3$	14.5	3.5
**12**	396.2627	396.2646	$C_{24}H_{34}N_3O_2$	9.5	4.7
**13**	523.3643	523.3621	$C_{31}H_{47}N_4O_3$	10.5	4.1
**14**	571.3966	571.3953	$C_{31}H_{51}N_6O_4$	9.5	2.3
**15**	585.4123	585.4113	$C_{32}H_{53}N_6O_4$	9.5	1.6
**16**	599.4279	599.4266	$C_{33}H_{55}N_6O_4$	9.5	2.1
**17**	613.4409	613.4418	$C_{34}H_{57}N_6O_4$	9.5	2.8
**18**	274.1914	274.1900	$C_{16}H_{24}N_3O$	6.5	5.1
**19**	472.3282	472.3279	$C_{26}H_{42}N_5O_3$	8.5	0.6
**20**	472.3282	472.3269	$C_{26}H_{42}N_5O_3$	8.5	2.8
**21**	486.3439	486.3435	$C_{27}H_{44}N_5O_3$	8.5	0.7
**22**	500.3595	500.3597	$C_{28}H_{46}N_5O_3$	8.5	-0.3
**23**	373.2598	373.2595	$C_{21}H_{33}N_4O_2$	7.5	0.8
**24**	571.3966	571.3957	$C_{31}H_{51}N_6O_4$	9.5	1.7
**25**	585.4123	585.4110	$C_{32}H_{53}N_6O_4$	9.5	2.2
**26**	599.4279	599.4266	$C_{33}H_{55}N_6O_4$	9.5	2.2
**27**	486.3439	486.3431	$C_{27}H_{44}N_5O_3$	8.5	0.5
**28**	288.2070	288.2071	$C_{17}H_{26}N_3O$	6.5	-0.2
**29**	322.1914	322.1911	$C_{20}H_{24}N_3O$	10.5	0.8
**30**	520.3282	520.3275	$C_{30}H_{42}N_5O_3$	12.5	1.4
**31**	534.3439	534.3433	$C_{31}H_{44}N_5O_3$	12.5	1.1
**32**	548.3595	548.3583	$C_{32}H_{46}N_5O_3$	12.5	2.2
**33**	401.2911	401.2909	$C_{23}H_{37}N_4O_2$	7.5	0.4

**34**	585.4123	585.4112	$C_{32}H_{53}N_6O_4$	9.5	1.9
**35**	585.4123	585.4112	$C_{32}H_{53}N_6O_4$	9.5	1.9
**36**	599.4279	599.4264	$C_{33}H_{55}N_6O_4$	9.5	2.5
**37**	633.4123	633.4104	$C_{36}H_{53}N_6O_4$	13.5	3.0
**38**	681.4279	695.4263	$C_{41}H_{55}N_6O_4$	17.5	2.4
**39**	633.4123	633.4104	$C_{36}H_{53}N_6O_4$	13.5	3.0
**40**	647.4279	647.4262	$C_{37}H_{55}N_6O_4$	13.5	2.7
**41**	737.4749	737.4714	$C_{44}H_{61}N_6O_4$	17.5	4.7
**42**	723.4598	723.3896	$C_{43}H_{59}N_6O_4$	17.5	5.0
**43**	647.4279	647.4365	$C_{37}H_{55}N_6O_4$	13.5	3.8
**44**	557.3810	557.3804	$C_{30}H_{49}N_6O_4$	9.5	1.1
**45**	571.3966	571.3955	$C_{31}H_{51}N_6O_4$	9.5	2.0
**46**	458.3126	458.3120	$C_{25}H_{40}N_5O_3$	8.5	1.3
**47**	472.3282	472.3278	$C_{26}H_{42}N_5O_3$	8.5	0.9
**48**	585.4123	585.4112	$C_{32}H_{53}N_6O_4$	9.5	1.9
**49**	359.2442	359.2439	$C_{20}H_{31}N_4O_2$	7.5	0.6

Table S2. Relative production of RXPs in different strains, related to Figure 2. Production of compound **1** was normalized as 100 ± 10.0 (means ± SD), all the rest RXPs were calculated based on compound **1**. The data is shown for n = 3.

RXP	Structure	Relative amount							
		a	b	c	d	e	f	g	h
**1**	mL-mL-mL-PEA	100 ± 10.0	0.4 ± 0.06	–	–	–	–	–	–
**2**	L-mL-PEA	0.4 ± 0.1	–	–	–	–	–	–	–
**3**	mL-mL-PEA	0.3 ± 0.04	–	0.2 ± 0.03	–	–	–	–	–
**4**	mL-L-L-PEA	1.9 ± 0.2	–	–	–	–	–	–	–
**5**	mL-mL-L-PEA	28.4 ± 0.9	–	–	–	–	–	–	–
**6**	mF-mF-PEA	–	–	–	8.0 ± 0.4	–	0.8 ± 0.04	1.0 ± 0.7	–
**7**	mF-mF-mF-PEA	–	–	–	8.4 ± 0.3	–	0.5 ± 0.04	2.7 ± 0.4	–
**8**	mF-mF-mL-PEA	–	–	–	–	0.4 ± 0.03	–	–	–
**9**	mL-mF-PEA	–	–	–	–	–	9.4 ± 0.3	–	–
**10**	mL-F-mF-PEA	–	–	–	–	–	1.0 ± 0.1	–	–
**11**	mL-mF-mF-PEA	–	–	–	–	–	0.07 ± 0.01	–	–
**12**	mV-mF-PEA	–	–	–	–	–	–	10.6 ± 0.1	–
**13**	mV-mF-mL-PEA	–	–	–	–	–	–	–	0.2 ± 0.03

Table S3. Bacterial strains used in this study.

Strain	Relevant Genotype	Reference/Strain No.
E. coli		
DH10B MtaA	F– *mcr*A, Δ(*mrr*-*hsd*RMS-*mcr*BC), Φ80*lac*ZΔM15, Δ*lac*X74, *rec*A1, *end*A1, *ara*D139, Δ(*ara leu*)7697, *gal*U, *gal*K, *rps*L, *nup*G, λ–, *entD::mtaA*	[1,2]
Xenorhabdus		
X. nematophila	Wild type	ATCC 19601
KJ12.1	Wild type	[3]
X. innexi	Wild type	DSM 16336
X. vietnamensis	Wild type	DSM 22392
X. cabanilasii	Wild type	DSM 17905 [4]

DSM: Deutsche Sammlung von Mikroorganismen und Zellkulturen; ATCC: American Type Culture Collection.

Table S4. Primers used in this study.

Primer	Sequence (5'-3')	Targeting DNA fragment	Plasmid
XC117-Fw	ATGAAAAATGCTGCGCAAATTGTTAATG	*vietC* from *X. vietnamensis* (5,970 bp)	pCX99
XC92-Rv	TTAGTTATAAAATTGACTGATTCTCTC		
XC93-Fw	AAGAGAGAATCAGTCAATTTTATAACTAACAATTAATCATCGGCTCGTATAATG	pCDF-ara-tacI vector backbone (3,421 bp)	
XC115-Rv2	ATTAACAATTTGCGCAGCATTTTTCATGGAATTCCTCCTGTTAGCCCAAAAAAAC		
XC92-Fw1	ATGAAAAATGCCACGCAAATTATCAATGAG	*vietA* from *X. vietnamensis* (4,044 bp)	pCX106
XC122-Rv	TCATATAGCACCTTTCAATAGTTTTTG		
XC93-Fw2	AAGAGAGAATCAGTCAATTTTATAACTAACAATTAATCATCGGCTCGTATAATG	pCOLA-ara-tacI vector backbone (3,363 bp)	
XC93-Rv1	ATTGATAATTTGCGTGGCATTTTTCATGGAATTCCTCCTGTTAGCCCAAAAAAAC		
XC92-Fw1	ATGAAAAATGCCACGCAAATTATCAATGAG	Partial sequence of *vietA* (1,025 bp)	pCX107
XC120-Rv	ATTTGTGGCCCATTCCATTTATGCAGCAACCATTCGTGAAGTAATGTATGTATTTC		
XC120-Fw	ATGGTTGCTGCATAAATGGAATGGGCCAC	DNA fragment encoding A-MT domain of *iInxA* from *X. innexi* (2,834 bp)	
XC121-Rv	AGGCTTGCCGGCGCAAATCACTTTCATTTGGGATAGGCAGCGCTTTTTTATC		
XC121-Fw	ATCCCAAATGAAAGTGATTTGCGCCGGCAAGCCTACG	The sequence after A-MT domain in *vietA* (339 bp)	
XC122-Rv	TCATATAGCACCTTTCAATAGTTTTTG		
XC93-Fw2	ACTCAAAAACTATTGAAAGGTGCTATATGACAATTAATCATCGGCTCGTATAATGTG	pCOLA-ara-tacI vector backbone (3,363 bp)	
XC93-Rv1	ATTGATAATTTGCGTGGCATTTTTCATGGAATTCCTCCTGTTAGCCCAAAAAAAC		
XC92-Fw2	ATGAAAAATGTCGCGCAAATTATCAATG	*vietB* from *X. vietnamensis* (4,044 bp)	pCX108
XC100-Rv	TCATATATCACCTTCCAATAGTTTTTGTGTCG		
XC93-Fw3	ACACAAAAACTATTGGAAGGTGATATATGACAATTAATCATCGGCTCGTATAATG	pACYC-ara-tacI vector backbone (3,807 bp)	
XC93-Rv2	ATTGATAATTTGCGCGACATTTTTCATGGAATTCCTCCTGTTAGCCCAAAAAAAC		
XC92-Fw2	ATGAAAAATGTCGCGCAAATTATCAATG	Partial sequence of *vietB* (1,620 bp)	pCX109
XC123-Rv	ATGTTCCTGCCGACCATTCCATTCATGCAGTAAGGTGTTTTTTTC		
XC122-Fw	ACCTTACTGCATGAATGGAATGGTCGGCAGGAACATGGCTTACAAG	DNA fragment encoding A-MT domain of *inxB* from *X. innexi* (2,796 bp)	
XC124-Rv	ATCATTTTCATTCGGGATTGGTAATGCTTTTTTATC		
XC123-Fw	AGCATTACCAATCCCGAATGAAAATGATTCGCGCCGACGAGTTTATATC	The sequence after A-MT domain in *vietB* (352 bp)	
XC100-Rv	TCATATATCACCTTCCAATAGTTTTTGTGTCG		
XC93-Fw3	ACACAAAAACTATTGGAAGGTGATATATGACAATTAATCATCGGCTCGTATAATG	pACYC-ara-tacI vector backbone (3,807 bp)	
XC93-Rv2	ATTGATAATTTGCGCGACATTTTTCATGGAATTCCTCCTGTTAGCCCAAAAAAAC		
XCLZ1-Fw1	ATGAAAAATGCAGCGCAAATTG		
XCLZ1-Rv2	TGCAAGCGGCATTCCAACCAG	*cabAB* from *X.*	

XCLZ1-Fw2<br>XCLZ1-Rv1	ATATTCTGGTTGGAATGCCGC<br>TCATATATCACCTTTCAATAG	*cabanilasii* DSM 17905 (8,243 bp)	pCXLZ1
XCLZ2-Fw<br><br>XCLZ2-Rv	ACAAGAACTATTGAAAGGTGATATATGAC AATTAATCATCGGCTCGTATAATG<br>TCATTCACAATTTGCGCTGCATTTTTCATG GAATTCCTCCTGTTAGC	pCOLA-ara-tacI vector backbone (3,368 bp)	
XCLZ3-Fw1<br>XCLZ3-Rv2<br>XCLZ3-Fw2<br>XCLZ3-Rv1	ATGAAAAATGCCGCGCAGATC<br>TTCTGTTTCTTGCCCAACCTGGTAATTG<br>ACATTTCCAGGTTGGGCAAGAAACAGAAC<br>TGATTAATTGTCATATATCACCTTTCAG	*cabBC from X. cabanilasii* DSM 17905 (9,428 bp)	pCXLZ2
XCLZ4-Fw<br><br>XCLZ4-Rv	ACTGAAAGGTGATATATGACAATTAAT CATCGGCTCGTATAATG<br>TCATTGATGATCTGCGCGGCATTTTTCAT GGAATTCCTCCTGTTAGC	pACYC-ara-tacI vector backbone (3,900 bp)	
XCLZ5-Fw1<br>XCLZ5-Rv2<br>XCLZ5-Fw2<br>XCLZ5-Rv1	ATGAAAAATGCCGCGCAGATCATC<br>ATTGGTTGGCTTGTTCATTCAG<br>TCAAGCCCTGAATGAACAAGCC<br>TGTTAATCATAAAACTGACTGATAC	*cabCD from X. cabanilasii* DSM 17905 (10,733 bp)	pCXLZ3
XCLZ6-Fw<br><br>XCLZ6-Rv	AGAGAGTATCAGTCAGTTTTATGATTAAC AATTAATCATCGGCTCGTATAATG<br>CATTGATGATCTGCGCGGCATTTTTCATG GAATTCCTCCTGTTAGC	pCDF-ara-tacI vector backbone (3,421 bp)	
XCLZ7-Fw<br>XCLZ7-Rv	ATGAAAAATGCTGTGCAAATTGTC<br>TTAATCATAAAACTGACTGATAC	*cabD from X. cabanilasii* DSM 17905 (6,027 bp)	pCXLZ4
XCLZ8-Fw<br><br>XCLZ8-Rv	AGAGAGTATCAGTCAGTTTTATGATTAAC AATTAATCATCGGCTCGTATAATG<br>TCATTGACAATTTGCACAGCATTTTTCATG GAATTCCTCCTGTTAGC	pCDF-ara-tacI vector backbone (3,421 bp)	
XCLZ1-Fw1<br>XCLZ9-Rv	ATGAAAAATGCAGCGCAAATTG<br>TGTTCATTCAATGCCTGATAATTCAATGAA TTGTTGC	Part of *cabA from X. cabanilasii* DSM 17905 (1,745 bp)	pCXLZ5
XCLZ9-Fw<br><br>XCLZ10-Rv	TCATTGAATTATCAGGCATTGAATGAACA GGCCAAC<br>ACTCAATCCTGAACCCTCTCACCTTCACT TGGGTATCAAG	DNA fragment encoding A domain from *vietB* (1,144 bp)	
XCLZ10-Fw<br><br>XCLZ11-Rv	AGTGAAGGTGAGAGGGTTCAGGATTGAG TTAG<br>TCATATATCACCTTCCAATAG	The rest part of cabA from *X. cabanilasii* DSM 17905 (694 bp)	
XCLZ11-Fw<br><br>XCLZ2-Rv	ACAAGAACTATTGGAAGGTGATATATGAC AATTAATCATCGGCTCGTATAATG<br>TCATTCACAATTTGCGCTGCATTTTTCATG GAATTCCTCCTGTTAGC	pCOLA-ara-tacI vector backbone (3,368 bp)	
XCLZ1-Fw1<br>XCLZ12-Rv	ATGAAAAATGCAGCGCAAATTG<br>AGGGCTTGATAATTCAATGAATTGTTGC	Part of *cabA from X. cabanilasii* DSM 17905 (1,736 bp)	pCXLZ6
XCLZ12-Fw<br><br>XCLZ13-Rv	ACAATTCATTGAATTATCAAGCCCTGAAT GAACAGGCCAATC<br>ACCCTCGGATTTTGACCTGAGAATCCAG	DNA fragment encoding A domain from *inxB* (1,144 bp)	
XCLZ13-Fw<br><br>XCLZ11-Rv	TGGATTCTCAGGTCAAAATCCGAGGGTTC AGGATTGAGTTAGG<br>TCATATATCACCTTCCAATAG	The rest part of cabA from *X. cabanilasii* DSM 17905 (703 bp)	
XCLZ11-Fw<br><br>XCLZ2-Rv	ACAAGAACTATTGGAAGGTGATATATGAC AATTAATCATCGGCTCGTATAATG<br>TCATTCACAATTTGCGCTGCATTTTTCATG GAATTCCTCCTGTTAGC	pCOLA-ara-tacI vector backbone (3,368 bp)	
XCLZ1-Fw1<br>XCLZ12-Rv	ATGAAAAATGCAGCGCAAATTG<br>AGGGCTTGATAATTCAATGAATTGTTGC	Part of *cabA from X. cabanilasii* DSM 17905 (1,736 bp)	

XCLZ12-Fw	ACAATTCATTGAATTATCAAGCCCTGAATGAACAGGCCAATC	DNA fragment encoding A-MT domain from *inxB* (2,522 bp)	pCXLZ7
XCLZ14-Rv	AGCTGAGTATTGCGGATAATTTGCCAAATAGGTTTG		
XCLZ14-Fw	TGGCAAATTATCCGCAATACTCAGCTATTAAAGCTG	The rest part of cabA from *X. cabanilasii* DSM 17905 (509 bp)	
XCLZ11-Rv	TCATATATCACCTTCCAATAG		
XCLZ11-Fw	ACAAGAACTATTGGAAGGTGATATATGACAATTAATCATCGGCTCGTATAATG	pCOLA-ara-tacI vector backbone (3,368 bp)	
XCLZ2-Rv	TCATTCACAATTTGCGCTGCATTTTTCATGGAATTCCTCCTGTTAGC		
XCLZ3-Fw1	ATGAAAAATGCCGCGCAGATC	Part of *cabBC from X. cabanilasii* DSM 17905 (4,695 bp)	pCXLZ8
XCLZ3-Rv2	TTCTGTTTCTTGCCCAACCTGGTAATTG		
XCLZ3-Fw2	ACATTTCCAGGTTGGGCAAGAAACAGAAC	Part of *cabC from X. cabanilasii* DSM 17905 (4,695 bp)	
XCLZ15-Rv	TGGCCTGTTCATTCAATGCCTGATAACTTAAAGCCTCTC		
XCLZ15-Fw	TGGATTCTCAGGTCAAAATCCGAGGGTTCAGGATTGAGTTAGG	DNA fragment encoding A-MT domain from *vietB* (1,795 bp)	
XCLZ16-Rv	TCATATATCACCTTCCAATAG		
XCLZ16-Fw	TGGCTAATTATCCCCAACTTCCTGCTATTAGCGGAG	The rest part of *cabC* from *X. cabanilasii* DSM 17905 (503 bp)	
XCLZ3-Rv1	TGATTAATTGTCATATATCACCTTTCAG		
XCLZ4-Fw	ACTGAAAGGTGATATATGACAATTAATCATCGGCTCGTATAATG	pACYC-ara-tacI vector backbone (3,900 bp)	
XCLZ4-Rv	TCATTGATGATCTGCGCGGCATTTTTCATGGAATTCCTCCTGTTAGC		
XCLZ3-Fw1	ATGAAAAATGCCGCGCAGATC	Part of *cabB from X. cabanilasii* DSM 17905 (1,745 bp)	pCXLZ9
XCLZ17-Rv	AGGGCTTGATAATTCAATGATTGCCAG		
XCLZ17-Fw	ACTGGCAATCATTGAATTATCAAGCCCTGAATGAACAGGCCAATC	DNA fragment encoding A-MT domain from *inxB* (2,522 bp)	
XCLZ18-Rv	TGCCAAATAGGTTTGGCTATAATG		
XCLZ18-Fw2	AGGCGCATTATAGCCAAACCTATTTGGCAAATTATCCGCAAATTTCGGCTATTAG	The rest part of *cabBC* from *X. cabanilasii* DSM 17905 (5,212 bp)	
XCLZ3-Rv1	TGATTAATTGTCATATATCACCTTTCAG		
XCLZ4-Fw	ACTGAAAGGTGATATATGACAATTAATCATCGGCTCGTATAATG	pACYC-ara-tacI vector backbone (3,900 bp)	
XCLZ4-Rv	TCATTGATGATCTGCGCGGCATTTTTCATGGAATTCCTCCTGTTAGC		
XCLZ7-Fw	ATGAAAAATGCTGTGCAAATTGTC	Part of *cabD from X. cabanilasii* DSM 17905 (1,745 bp)	pCXLZ10
XCLZ19-Rv	TGGCCTGTTCATTCAATGCCTGATAGCTTAAAGTACTGTCAC		
XCLZ19-Fw	AGCTATCAGGCATTGAATGAACAGGCCAAC	DNA fragment encoding A-MT domain from *vietB* (2,490 bp)	
XCLZ20-Rv	TGCCAAATAGGTTTGGCTATAATG		
XCLZ20-Fw	ACCCATTTGGCTAATTATCCCCAACTCCCTGCTATTAGCGGAG	The rest part of *cabD* from *X. cabanilasii* DSM 17905 (1,839 bp)	
XCLZ7-Rv	TTAATCATAAAACTGACTGATAC		
XCLZ8-Fw	AGAGAGTATCAGTCAGTTTTATGATTAACAATTAATCATCGGCTCGTATAATG	pCDF-ara-tacI vector backbone (3,421 bp))	
XCLZ8-Rv	TCATTGACAATTTGCACAGCATTTTTCATGGAATTCCTCCTGTTAGC		
XCLZ7-Fw	ATGAAAAATGCTGTGCAAATTGTC	Part of *cabD* from *X. cabanilasii* DSM 17905 (1,745 bp)	
XCLZ21-Rv	ACTGATTGGCCTGTTCATTCAGGGCTTGATAG		

XCLZ21-Fw<br>XCLZ22-Rv	AGCCCTGAATGAACAGGCCAATCAGTTAGCCCGTTATC<br>TGCGGATAATTTGCCAAATAGGTTTGGCTATAATG	DNA fragment encoding A-MT domain from *inxB* (2,493 bp)	pCXLZ11
XCLZ22-Fw<br>XCLZ7-Rv	AGCCAAACCTATTTGGCAAATTATCCGCAACTCCCTGC<br>TTAATCATAAAACTGACTGATAC	The rest part of *cabD* from *X. cabanilasii* DSM 17905 (1,839 bp)	
XCLZ8-Fw<br>XCLZ8-Rv	AGAGAGTATCAGTCAGTTTTATGATTAACAATTAATCATCGGCTCGTATAATG<br>TCATTGACAATTTGCACAGCATTTTTCATGGAATTCCTCCTGTTAGC	pCDF-ara-tacI vector backbone (3,421 bp)	
XCLZ1-Fw1<br>XCLZ23-Rv	ATGAAAAATGCAGCGCAAATTG<br>ACGGTTGAGTTCACCGTAATTCAATGAATTGTTGCCAAAAAC	Part of *cabA* from *X. cabanilasii* DSM 17905 (1,745 bp)	pCXLZ12
XCLZ23-Fw<br>XCLZ24-Rv	TCATTGAATTACGGTGAACTCAACCGTCGTGCC<br>ACTCAATCCTGAAACCCCGCAGTTTGACCTGAAAATC	DNA fragment encoding A domain (Thr) from xenoamicin gene cluster (1,186 bp)	
XCLZ24-Fw<br>XCLZ11-Rv	AGGTCAAACTGCGGGGTTTCAGGATTGAGTTAGGAGAAATC<br>TTAATCATAAAACTGACTGATAC	The rest part of *cabA* from *X. cabanilasii* DSM 17905 (1,839 bp)	
XCLZ11-Fw<br>XCLZ2-Rv	ACAAGAACTATTGGAAGGTGATATATGACAATTAATCATCGGCTCGTATAATG<br>TCATTCACAATTTGCGCTGCATTTTTCATGGAATTCCTCCTGTTAGC	pCOLA-ara-tacI vector backbone (3,368 bp)	
XCLZ7-Fw<br>XCLZ25-Rv	ATGAAAAATGCTGTGCAAATTGTC<br>ACGATTGGCGCAGCGGTTCAGTTCACCATAGCTTAAAGTACTGTCACCAAAG	Part of *cabD* from *X. cabanilasii* DSM 17905 (1,745 bp)	pCXLZ13
XCLZ25-Fw<br>XCLZ26-Rv	ATGGTGAACTGAACCGCTGCG<br>ATGCGATGGCTGCGGATCTTGACCTGAAAATCATTACGAC	DNA fragment encoding A domain (Ala) from xenoamicin gene cluster (1,189 bp)	
XCLZ26-Fw<br>XCLZ7-Rv	AGGTCAAGATCCGCAGCCATCGCATTGAAC<br>TTAATCATAAAACTGACTGATAC	The rest part of *cabD* from *X. cabanilasii* DSM 17905 (1,839 bp)	
XCLZ8-Fw<br>XCLZ8-Rv	AGAGAGTATCAGTCAGTTTTATGATTAACAATTAATCATCGGCTCGTATAATG<br>TCATTGACAATTTGCACAGCATTTTTCATGGAATTCCTCCTGTTAGC	pCDF-ara-tacI vector backbone (3,421 bp)	
XCLZ3-Fw1<br>XCLZ17-Rv	ATGAAAAATGCCGCGCAGATC<br>AGGGCTTGATAATTCAATGATTGCCAG	Part of *cabB from X. cabanilasii* DSM 17905 (1,745 bp)	pCXLZ14
XCLZ17-Fw<br>XCLZ18-Rv	ACTGGCAATCATTGAATTATCAAGCCCTGAATGAACAGGCCAATC<br>TGCCAAATAGGTTTGGCTATAATG	DNA fragment encoding A-MT domain from *inxB* (2,504 bp)	
XCLZ18-Fw2<br>XCLZ3-Rv1	AGGCGCATTATAGCCAAACCTATTTGGCAAATTATCCGCAAATTTCGGCTATTAG<br>TGATTAATTGTCATATATCACCTTTCAG	The rest part of *cabB* from *X. cabanilasii* DSM 17905 (532 bp)	
XCLZ2-Fw<br>XCLZ4-Rv	ACAAGAACTATTGAAAGGTGATATATGACAATTAATCATCGGCTCGTATAATG<br>TCATTGATGATCTGCGCGGCATTTTTCATGGAATTCCTCCTGTTAGC	pACYC-ara-tacI vector backbone (3,900 bp)	
XCLZ5-Fw1<br>XCLZ27-Rv	ATGAAAAATGCCGCGCAGATCATC<br>ATTCAATGCCTGATAACTTAAAGCCTCTC	Part of *cabCD from X. cabanilasii* DSM 17905 (1,734 bp)	

XCLZ27-Fw	TGGAGAGGCTTTAAGTTATCAGGCATTGAATGAACAGG	DNA fragment encoding A-MT domain from *vietB* (2,506 bp)	
XCLZ28-Rv	AGCAGGAAGTTGGGGATAATTAGCCAAATGGGTC		
XCLZ28-Fw	TGGCTAATTATCCCCAACTTCCTGCTATTAGCGGAG	The rest part of *cabD* from *X. cabanilasii* DSM 17905 (6,525 bp)	pCXLZ15
XCLZ5-Rv1	TGTTAATCATAAAACTGACTGATAC		
XCLZ6-Fw	AGAGAGTATCAGTCAGTTTTATGATTAACAATTAATCATCGGCTCGTATAATG	pCDF-ara-tacI vector backbone (3,421 bp)	
XCLZ6-Rv	CATTGATGATCTGCGCGGCATTTTTCATGGAATTCCTCCTGTTAGC		
XCLZ3-Fw1	ATGAAAAATGCCGCGCAGATC	Part of *cabBC* from *X. cabanilasii* DSM 17905 (6,842 bp)	
XCLZ29-Rv	TCATTCAGGGCTTGATAACTTAAAGCCTCTCCAC		
XCLZ29-Fw	AGAGGCTTTAAGTTATCAAGCCCTGAATGAACAGGCCAATC	DNA fragment encoding A-MT domain in *inxB* from *X. innexi* DSM 16336 (2,511 bp)	
XCLZ30-Rv	TGCGGATAATTTGCCAAATAGGTTTG		pCXLZ16
XCLZ30-Fw	AGCCAAACCTATTTGGCAAATTATCCGCAATTATCCGCAACTTCCTGC	The rest part of *cabC* from *X. cabanilasii* DSM 17905 (531 bp)	
XCLZ3-Rv1	TGATTAATTGTCATATATCACCTTTCAG		
XCLZ4-Fw	ACTGAAAGGTGATATATGACAATTAATCATCGGCTCGTATAATG	pACYC-ara-tacI vector backbone (3,900 bp)	
XCLZ4-Rv	TCATTGATGATCTGCGCGGCATTTTTCATGGAATTCCTCCTGTTAGC		
XCLZ5-Fw1	ATGAAAAATGCCGCGCAGATCATC	Part of *cabC from X. cabanilasii* DSM 17905 (1,734 bp)	
XCLZ27-Rv	ATTCAATGCCTGATAACTTAAAGCCTCTC		
XCLZ27-Fw	TGGAGAGGCTTTAAGTTATCAGGCATTGAATGAACAGG	DNA fragment encoding A-MT domain from *vietB* (2,506 bp)	
XCLZ28-Rv	AGCAGGAAGTTGGGGATAATTAGCCAAATGGGTC		
XCLZ28-Fw	TGGCTAATTATCCCCAACTTCCTGCTATTAGCGGAG	The rest part of *cabC* from *X. cabanilasii* DSM 17905 (499 bp)	pCXLZ17
XCLZ31-Rv	TGATTAATTGTCATATATCACCTTTCAGTATT		
XCLZ31-Fw	ATACTGAAAGGTGATATATGACAATTAATCATCGGCTCG	pCX27 vector backbone (3,421 bp)	
XCLZ6-Rv	CATTGATGATCTGCGCGGCATTTTTCATGGAATTCCTCCTGTTAGC		
XCLZ3-Fw1	ATGAAAAATGCCGCGCAGATC	DNA fragment of *cabB* from *X. cabanilasii* DSM 17905 with point mutation on MT coding region (3,250 bp)	
XCLZ32-Rv	ATACAACAATAAGCTGCTACTACAGCTAATTTCCAG		
XCLZ32-Fw	AGCTGTAGTAGCAGCTTATTGTTGTATCG	Rest part of *cabB* from *X. cabanilasii* DSM 17905 with point mutation on MT coding region (1,464 bp)	pCXLZ19
XCLZ1-Rv	TCATATATCACCTTTCAATAG		
XCLZ2-Fw	ACAAGAACTATTGAAAGGTGATATATGACAATTAATCATCGGCTCGTATAATG	pACYC-ara-tacI vector backbone (3,900 bp)	
XCLZ4-Rv	TCATTGATGATCTGCGCGGCATTTTTCATGGAATTCCTCCTGTTAGC		

XCLZ5-Fw1	ATGAAAAATGCCGCGCAGATCATC	DNA fragment of *cabC* from *X. cabanilasii* DSM 17905 with point mutation on MT coding region (3,263 bp)	pCXLZ20
XCLZ33-Rv	ACGCTTGTGCTACAGCTGATTTCCAATAAG		
XCLZ33-Fw	ATTGGAAATCAGCTGTAGCACAAGCGTTTTGTTGTA	Rest part of *cabC* from *X. cabanilasii* DSM 17905 with point mutation on MT coding region (1,474 bp)	
XCLZ31-Rv	TGATTAATTGTCATATATCACCTTTCAGTATT		
XCLZ31-Fw	ATACTGAAAGGTGATATATGACAATTAATCATCGGCTCG	pCX27 backbone (3,421 bp)	
XCLZ6-Rv	CATTGATGATCTGCGCGGCATTTTTCATGGAATTCCTCCTGTTAGC		
XCLZ7-Fw	ATGAAAAATGCTGTGCAAATTGTC	DNA fragment of *cabD* from *X. cabanilasii* DSM 17905 with point mutation on MT coding region (3,263 bp)	pCXLZ22
XCLZ34-Rv	ACAATAAGCTAGAGCTACAGCTGATTTCTAATAAGC		
XCLZ34-Fw	AGAAATCAGCTGTAGCTCTAGCTTATTGTTATATCG	Rest part of *cabD* from *X. cabanilasii* DSM 17905 with point mutations on MT coding region	
XCLZ7-Rv	TTAATCATAAAACTGACTGATAC		
XCLZ8-Fw	AGAGAGTATCAGTCAGTTTTATGATTAACAATTAATCATCGGCTCGTATAATG	pCDF-ara-tacI vector backbone (3,421 bp)	
XCLZ8-Rv	TCATTGACAATTTGCACAGCATTTTTCATGGAATTCCTCCTGTTAGC		
XCLZ1-Fw1	ATGAAAAATGCAGCGCAAATTG	DNA fragment of *cabA* from *X. cabanilasii* DSM 17905 (3,525 bp)	pCXLZ23
XCLZ11-Rv	TCATATATCACCTTCCAATAG		
XCLZ11-Fw	ACAAGAACTATTGGAAGGTGATATATGACAATTAATCATCGGCTCGTATAATG	pCOLA-ara-tacI vector backbone (3,421 bp)	
XCLZ2-Rv	TCATTCACAATTTGCGCTGCATTTTTCATGGAATTCCTCCTGTTAGC		
XCLZ5-Fw1	ATGAAAAATGCCGCGCAGATCATC	DNA fragment of *cabC* from *X. cabanilasii* DSM 17905 (4,710 bp)	pCXLZ24
XCLZ31-Rv	TGATTAATTGTCATATATCACCTTTCAGTATT		
XCLZ31-Fw	ATACTGAAAGGTGATATATGACAATTAATCATCGGCTCG	pACYC-ara-tacI vector backbone (3,421 bp)	
XCLZ6-Rv	CATTGATGATCTGCGCGGCATTTTTCATGGAATTCCTCCTGTTAGC		
XCLZ3-Fw1	ATGAAAAATGCCGCGCAGATC	DNA fragment of *cabB* from *X. cabanilasii* DSM 17905 (3,525 bp)	pCXLZ25
XCLZ1-Rv1	TCATATATCACCTTTCAATAG		
XCLZ2-Fw	ACAAGAACTATTGAAAGGTGATATATGACAATTAATCATCGGCTCGTATAATG	pACYC-ara-tacI vector backbone (3,421 bp)	
XCLZ4-Rv	TCATTGATGATCTGCGCGGCATTTTTCATGGAATTCCTCCTGTTAGC		

Table S5. Plasmids used in this study.

Plasmid	Expressed modules/Description	References
pCOLA-ara-tacI	modified from pCOLA_tacI/I that contains arabinose-inducible promotor and kanamycin resistance (Km^R) gene	[1]
pCDF-ara-tacI	modified from pCDF_tacI/I that contains arabinose-inducible promoter and spectinomycin resistance (Sm^R) gene	[1]
pACYC-ara-tacI	modified from pACYC_tacI/I contains arabinose-inducible promoter and chloramphenicol resistance (Cm^R) gene	[1]
pCX64	Kj12B(A-MT:InxB)-Kj12C; *kj12B* and *kj12C* from *Xenorhabdus* KJ12.1 with A-MT gene replaced by the second A-MT gene from *inxB* from *X. innexi* DSM 16336, assembled into pCX16 (Cm^R)	[1]
pCX26	Kj12B; *kj12B* from *Xenorhabdus* KJ12.1, assembled into pCX16, (Cm^R)	[1]
pCX19	Kj12C; *kj12C* from *Xenorhabdus* KJ12.1 assembled into pCDF-ara-tacI, (Sm^R)	[1]
pCX27	modified from pCOLA_tacI/I that contains arabinose-inducible promotor and apramycin resistance (Apr^R) gene	This study
pCX99	VietC; *vietC* from *X. vietnamensis* DSM 22392, assembled into pCDF-ara-tacI (Sm^R)	This study
pCX106	VietA; *vietA* from *X. vietnamensis* DSM 22392, assembled into pCOLA-ara-tacI (Km^R)	This study
pCX107	VietA (A-MT:InxA); *vietA* (A-MT:InxA), assembled into pCOLA-ara-tacI, Km^R	This study
pCX108	VietB; *vietB* from *X. vietnamensis* DSM 22392, assembled into pCX16 (Cm^R)	This study
pCX109	VietB (A-MT:InxB); *vietB* (A-MT:InxB), assembled into pCX16 (Cm^R)	This study
pCXLZ1	CabAB; *cabAB* from *X. cabanilanii* JM26, assembled into pCOLA-ara-tacI (Km^R)	This study
pCXLZ2	CabBC; *cabBC* from *X. cabanilanii* JM26, assembled into pACYC-ara-tacI (Cm^R)	This study
pCXLZ3	CabCD; *cabCD* from *X. cabanilanii* JM26, assembled into pCDF-ara-tacI (Sm^R)	This study
pCXLZ4	CabD; *cabD* from *X. cabanilanii* JM26, assembled into pCDF-ara-tacI (Sm^R)	This study
pCXLZ5	CabA (A:VietB); *cabA* from *X. cabanilanii* JM26 with A domain encoding region replaced by the one from *vietB*, assembled into pCOLA-ara-tacI (Km^R)	This study

Plasmid	Expressed modules/Description	References
pCXLZ6	CabA (A:InxB); *cabA* from *X. cabanilanii* JM26 with A domain encoding region replaced by the one from *inxB*, assembled into pCOLA-ara-tacI (Km^R)	This study
pCXLZ7	CabA (A-MT:InxB); *cabA* from *X. cabanilanii* JM26 with A domain encoding region replaced by the A-MT domain encoding region from *inxB*, assembled into pCOLA-ara-tacI (Km^R)	This study
pCXLZ8	CabB-CabC(A-MT:VietB); *cabBC* from *X. cabanilanii* JM26 with A–MT domain encoding region in CabC replaced by the one from *vietB*,	This study
pCXLZ9	CabB(A-MT:InxB)-CabC; *cabBC* from *X. cabanilanii* JM26 with A–MT domain encoding region in CabB replaced by the one from *inxB*,	This study
pCXLZ10	CabD (A-MT:VietB); *cabD* from *X. cabanilanii* JM26 with A-MT domain replaced by the one from *vietB*, assembled into pCDF-ara-tacI (Sm^R)	This study
pCXLZ11	CabD (A-MT:InxB); *cabD* from *X. cabanilanii* JM26 with A-MT domain replaced by the one from *inxB*, assembled into pCDF-ara-tacI (Sm^R)	This study
pCXLZ12	*cabA* from *X. cabanilanii* DSM 17905 with A domain encoding region replaced by the A domain encoding region from xenoamicin gene cluster for Thr specificity, assembled into pCOLA-ara-tacI (Km^R)	This study
pCXLZ13	*cabD* from *X. cabanilanii* DSM 17905 with A domain replaced by the one from xenoamicin gene cluster for Ala specificity, assembled into pCDF-ara-tacI (Sm^R)	This study
pCXLZ14	CabB (A-MT:InxB); *cabB* from *X. cabanilanii* JM26 with A–MT domain replaced by the one from *inxB*, assembled into pACYC-ara-tacI (Cm^R)	This study
pCXZL15	CabC(A-MT:VietB)-CabD; *cabCD* from *X. cabanilanii* JM26 with A-MT domain in CabC replaced by the one from *vietB*, assembled into pCDF-ara-tacI (Sm^R)	This study
pCXLZ16	CabB-CabC(A-MT:InxB); *cabBC* from *X. cabanilanii* JM26 with A-MT domain in CabC replaced by the one from *inxB*, assembled into pACYC-ara-tacI (Cm^R)	This study
pCXLZ17	CabC (A-MT:VietB); *cabBC* from *X. cabanilanii* JM26 with A-MT domain in CabC replaced by the one from *inxB*, assembled into pACYC-ara-tacI (Cm^R)	This study

Plasmid	Expressed modules/Description	References
pCXLZ19	CabB (MT-); *cabB* from *X. cabanilanii* JM26 with mutations on MT domain conserved motif (LLEIGCGSGLLL to LLEISCSSSLLL), assembled into pACYC-ara-tacI (Cm^R)	This study
pCXLZ20	CabC (MT-); *cabC* from *X. cabanilanii* JM26 with mutations on MT domain conserved motif (LLEIGCGSGLLL to LLEISCSSSLLL), assembled into pCX27 (Apr^R)	This study
pCXLZ22	CabD (MT-); *cabD* from *X. cabanilanii* JM26 with mutations on MT domain conserved motif (LLEIGCGSGLLL to LLEISCSSSLLL), assembled into pCDF-ara-tacI (Sm^R)	This study
pCXLZ23	CabA; *cabA* from *X. cabanilanii* JM26, assembled into pCOLA-ara-tacI (Km^R)	This study
pCXLZ24	CabC; *cabC* from *X. cabanilanii* JM26, assembled into pACYC-ara-tacI (Km^R)	This study
pCXLZ25	CabB; *cabB* from *X. cabanilanii* JM26, assembled into pACYC-ara-tacI (Km^R)	This study

Km^R: kanamycin resistance; Cm^R: chloramphenicol resistance; Sm^R: spectinomycin resistance; Apr^R: apramycin resistance.

Supplementary Figures

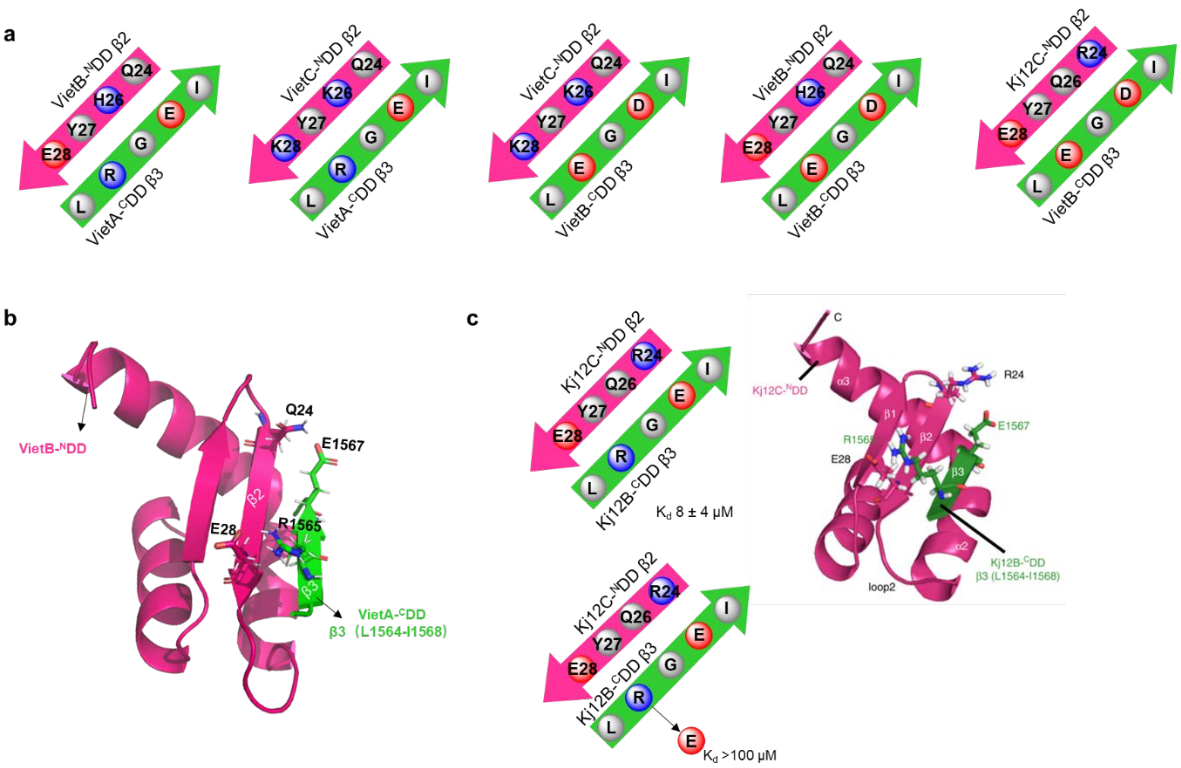

Figure S1. Docking domain (DD) interactions in VietABC system and VietB-^{C}DD/Kj12C-^{N}DD. a) Highlighting key residues in the interacting β-sheets according to previous study.[5,6] The interaction between ^{C}DD-^{N}DD relies on the salt bridges formed by a positively charged residue (blue circle) on the ^{N}DD and a negatively charged residue (red circle) on the ^{C}DD. b) The detailed structural view of interaction between VietB-^{N}DD and VietA-^{C}DD as an example, modelled by PyMOL based upon the structure of Kj12C-^{N}DD and Kj12B-^{C}DD published previously.[5] c) The model and structural view of DD interaction between Kj12C-^{N}DD and Kj12B-^{C}DD.[5]

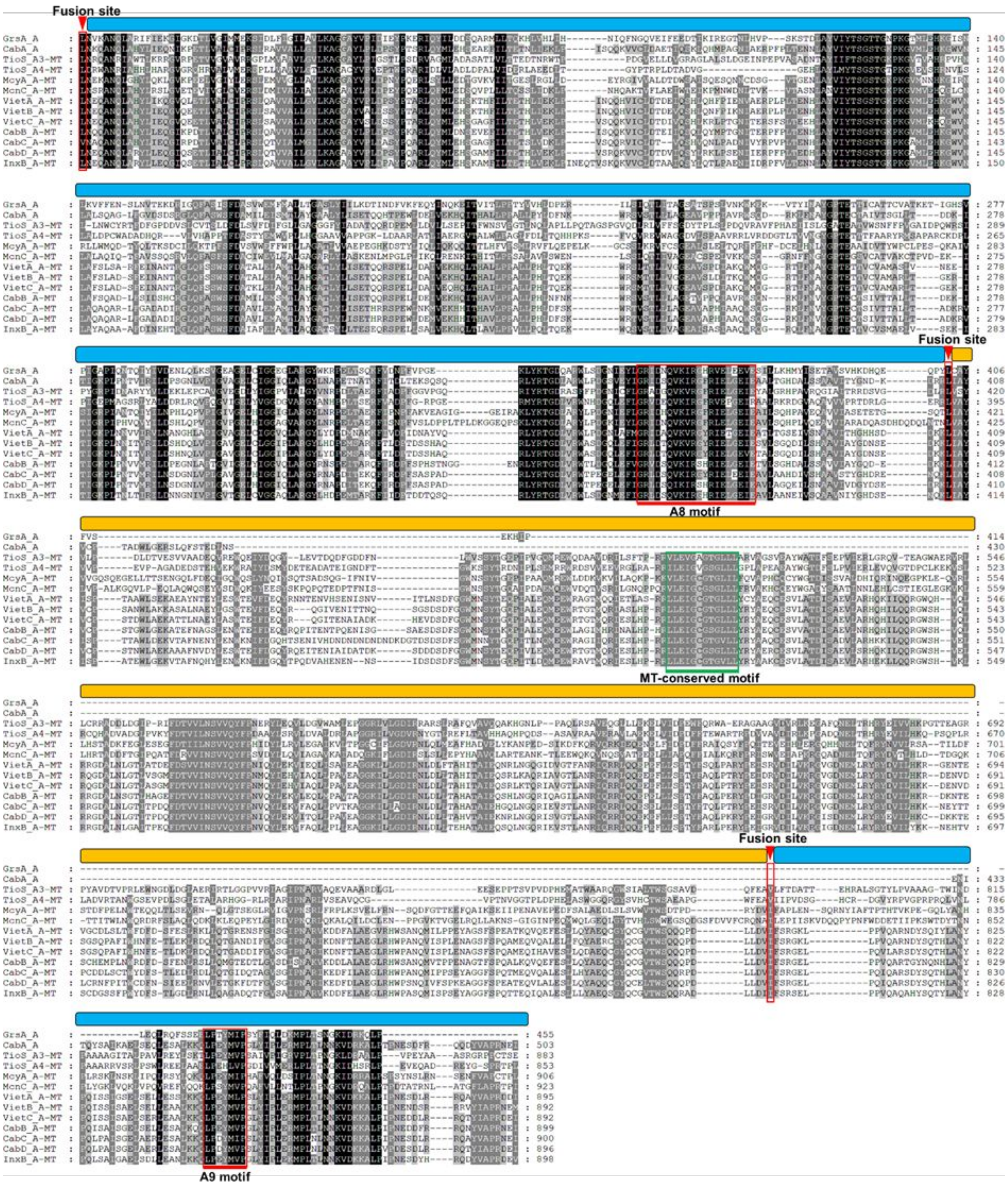

Figure S2. Sequence alignment of A and A-MT domains from selected classic NRPSs and RXP-NRPSs. Classic NRPSs, GrsA_A from gramicidin S synthetase (*Brevibacillus brevis*);[7] TioS_A3-MT and TioS_A4-MT from thiocoraline NRPS (*Micromonospora* sp. ML1);[8] McyA_A-MT from microcystin NRPS (*Microcystis aeruginosa* PCC 7806);[9] McnC_A-MT from micropeptin K139 (*Microcystis aeruginosa* NIES-298).[10] RXP-NRPSs, VietA_A-MT, VietB_A-MT and VietC_A-MT from VietABC (*X. vietamensis* DSM22392); CabA_A, CabB_A-MT, CabC_A-MT, and CabD_A-MT from CabABCD (*X. cabanilasii* DSM 17905); InxB_A-MT from InxABC (*X. innexi* DSM16336). (a) Overview of sequence alignment for A and A-MT domains from selected NRPSs, with indication of A domain (red bar), MT domain (yellow bar), A8, A9 and MT conserved motifs (purple bar, boxed with red dash line).[11] (b) The fusion sites used for A or A-MT domain exchange in this study.

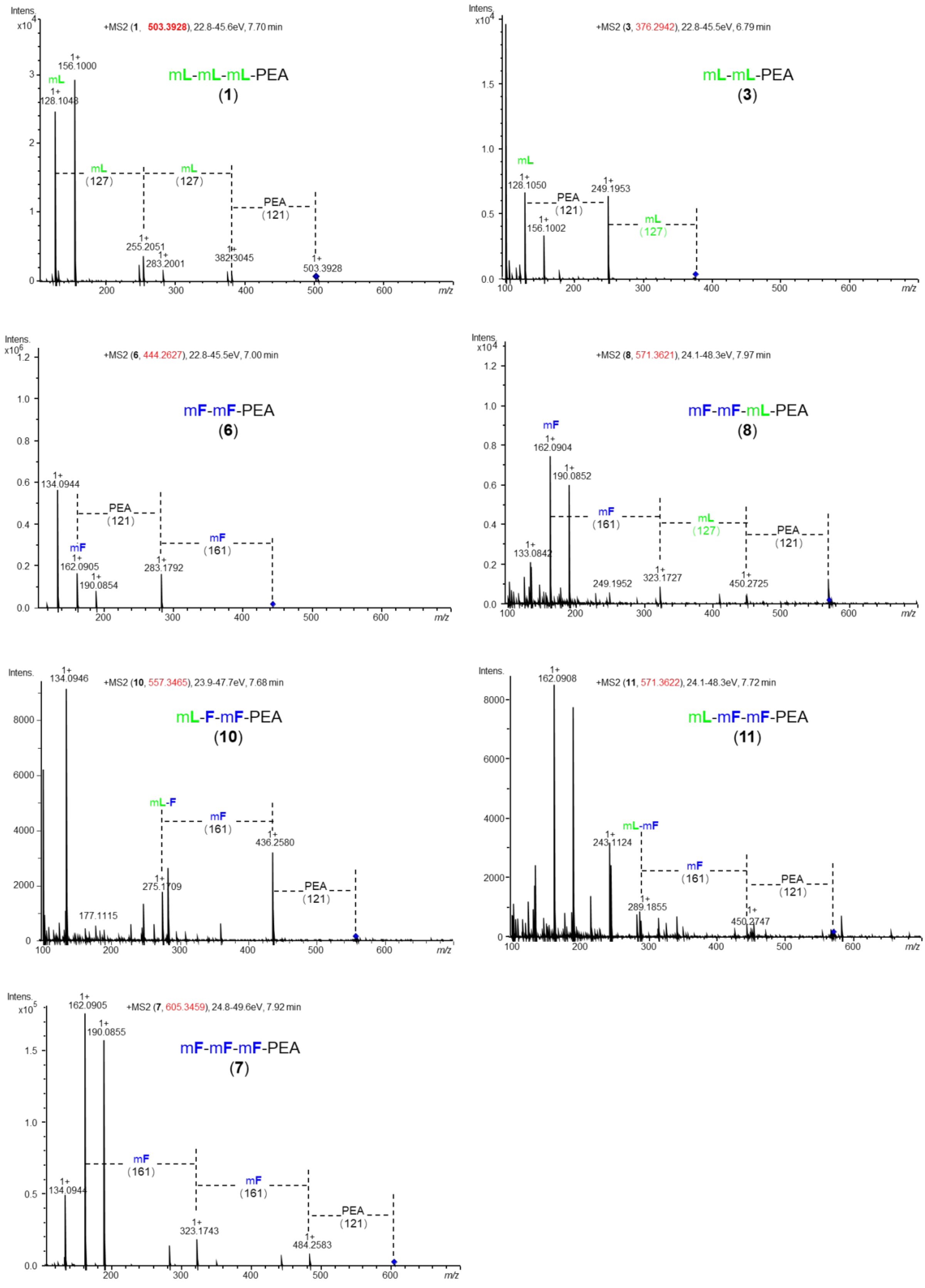

Figure S3. MS/MS analysis of RXPs **1**, **3**, **6**, **8**, **10–11** and **7**.

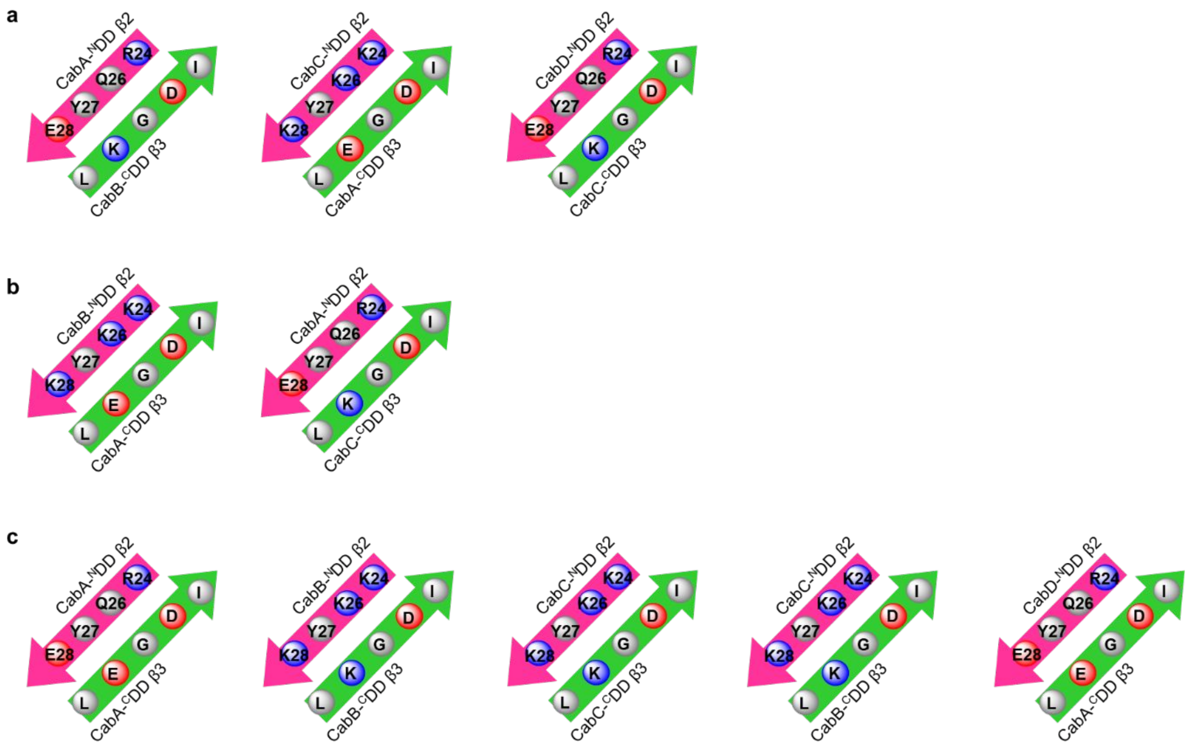

Figure S4. Docking domain (DD) interactions in CabABCD. Highlighting key residues in the interacting β-sheets according to previous study.[5,6] The interaction between ^{C}DD-^{N}DD relies on the salt bridges formed by a positively charged residue (blue circle) on the ^{N}DD and a negatively charged residue (red circle) on the ^{C}DD. (a) DD interactions that are observed in the order for the natural biosynthesis. (b) Other possibly strong DD interactions. (c) Additional and possibly weaker interactions between DDs in CabABCD system.

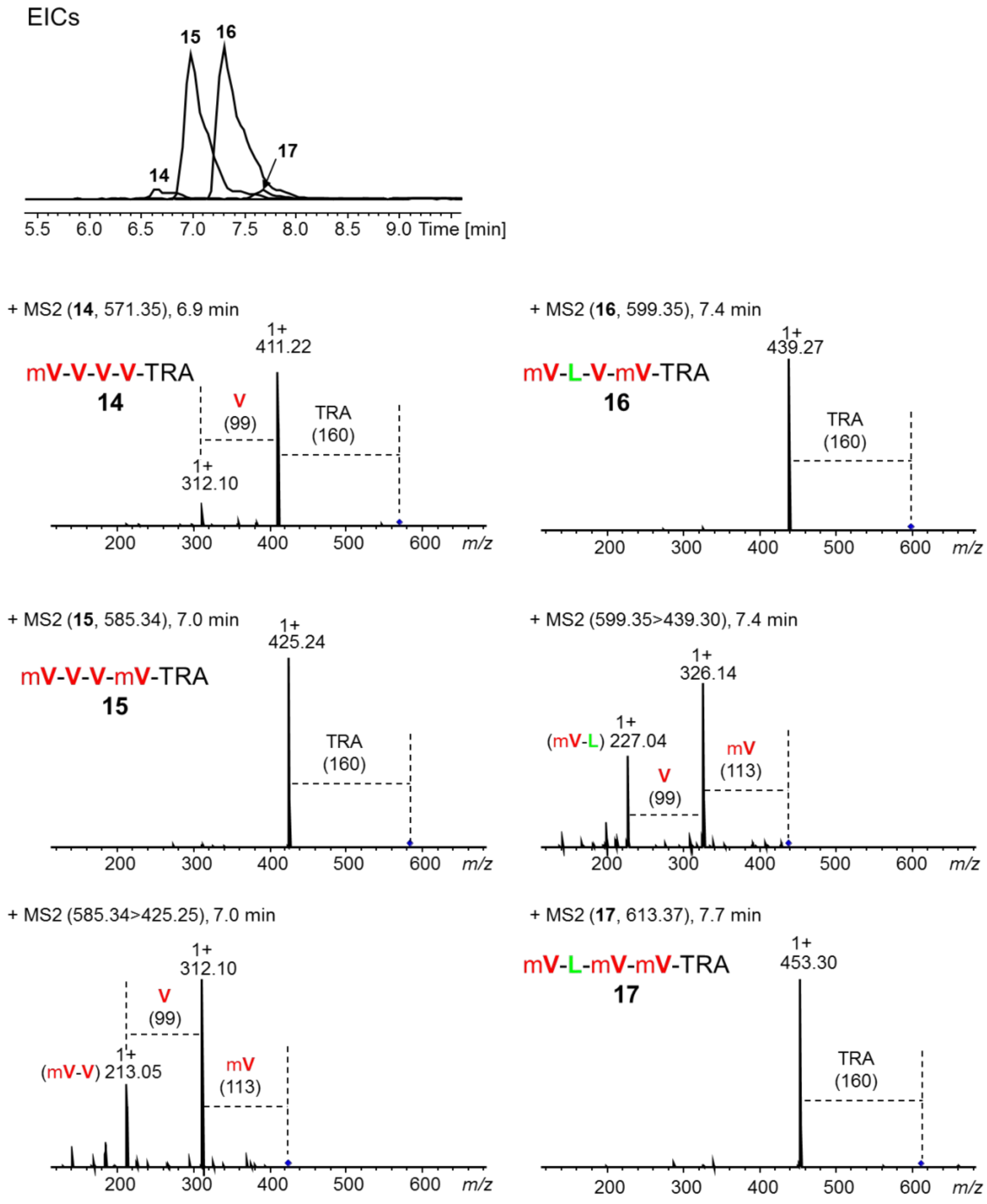

Figure S5. HPLC-MS/MS analysis of RXPs **14**–**17** produced in *X. cabanillasii* DSM 17905. EICs and MS^2 of **14**–**17** were shown.

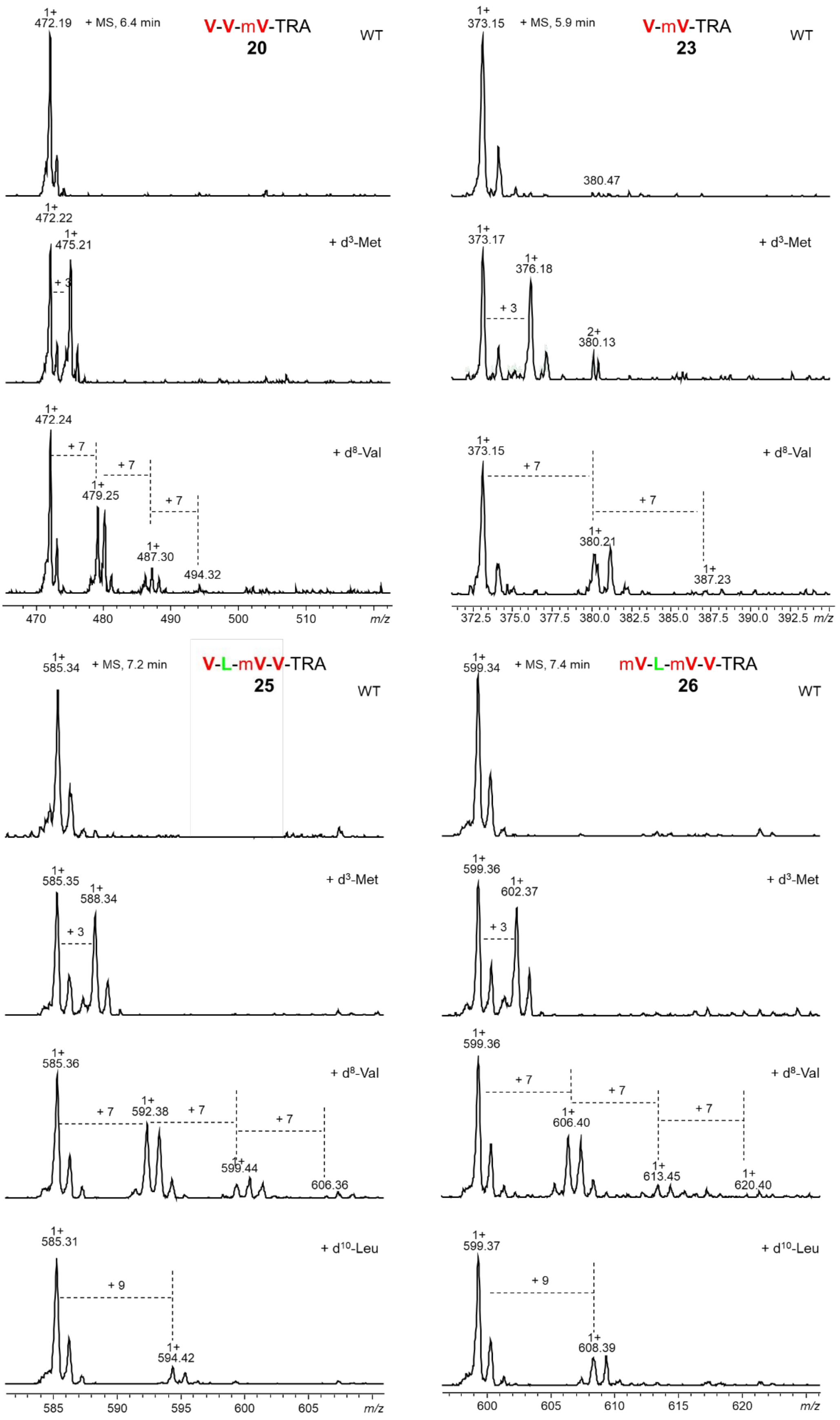

Figure S6. Selected examples of MS data from isotopic labeling experiments to elucidate the structures of RXPs in this study.

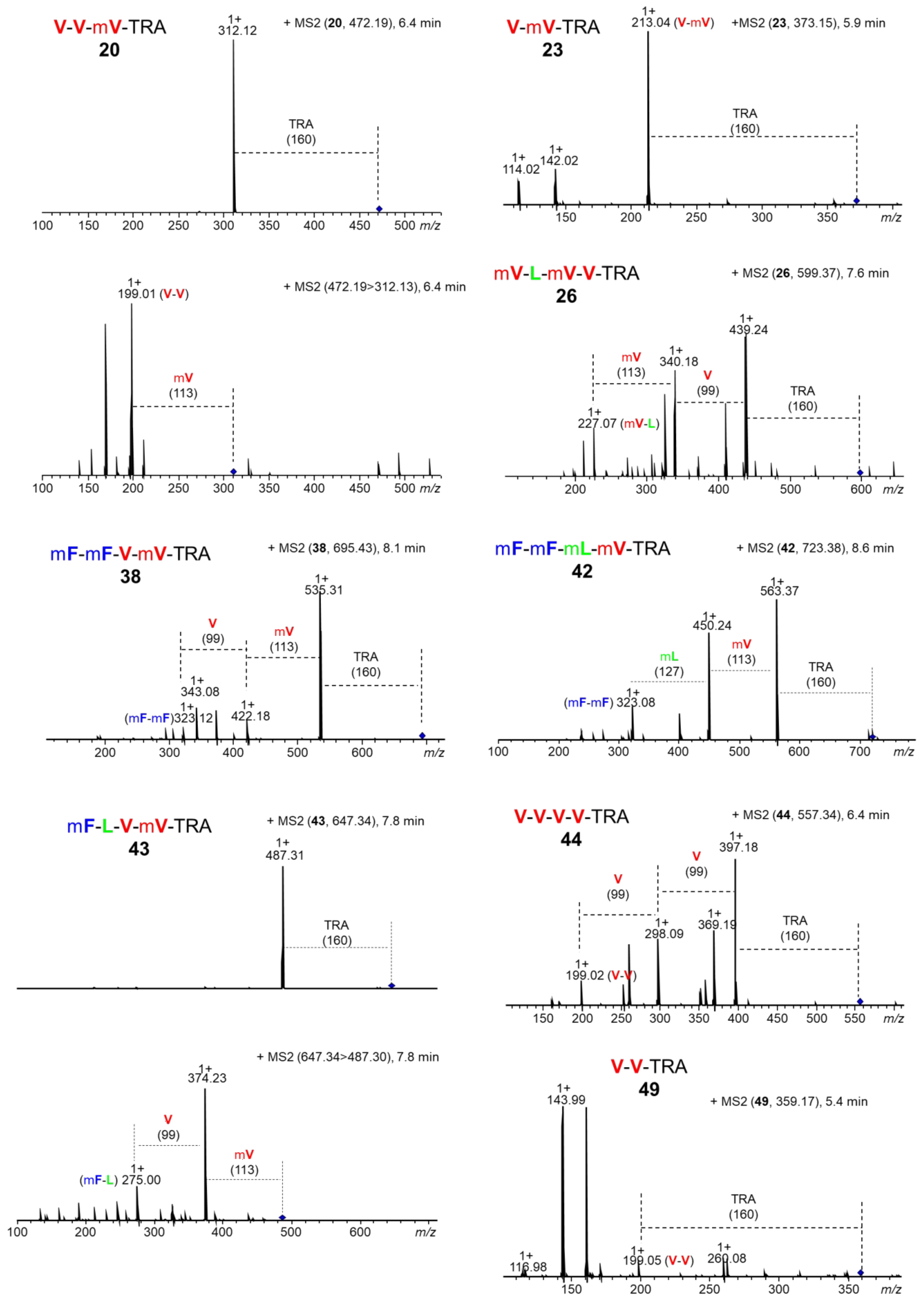

Figure S7. Structural elucidation of selected RXPs based on MS/MS fragmentations.

a

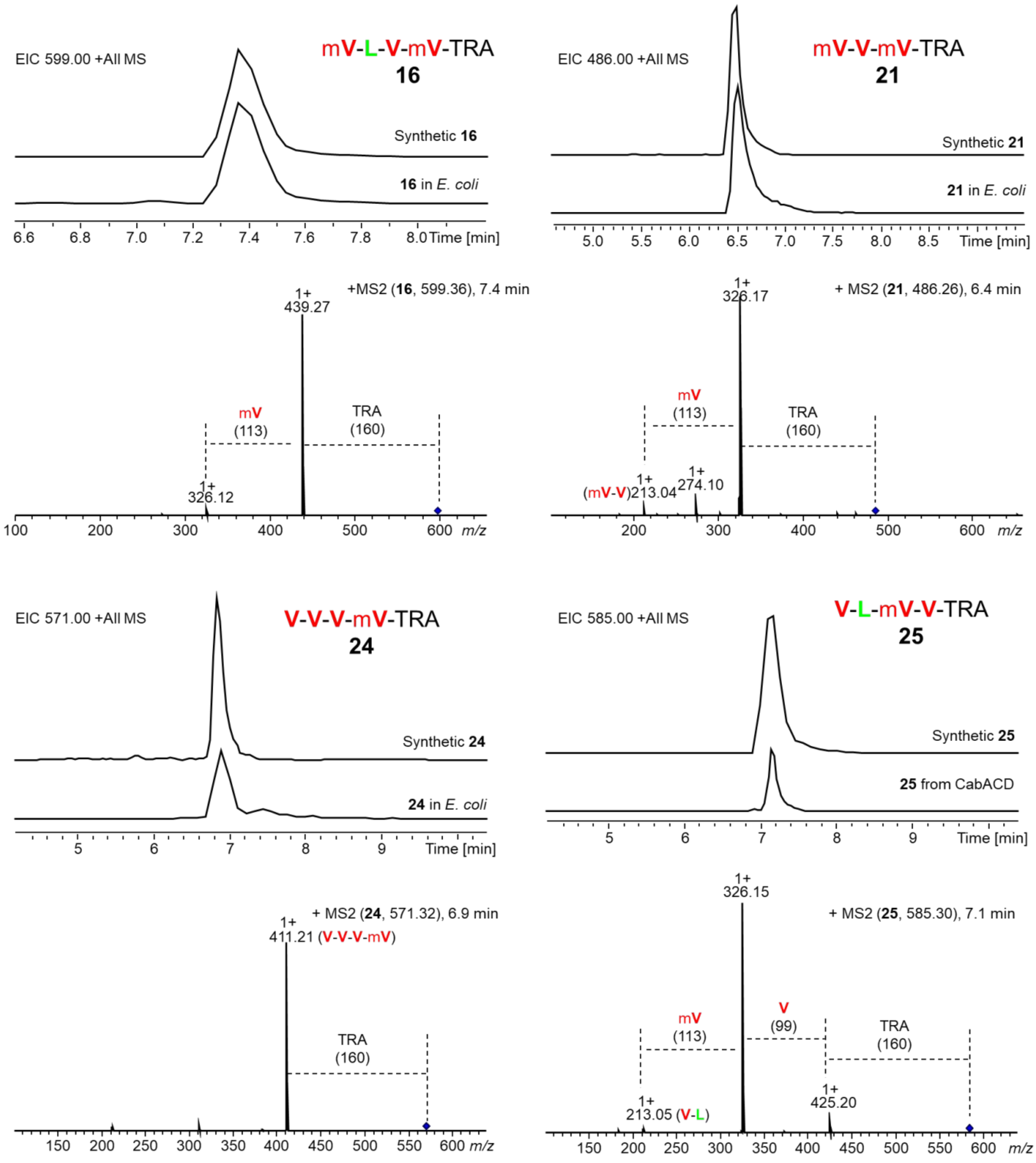

EIC 599.00 +All MS
mV-L-V-mV-TRA
16
Synthetic 16
16 in E. coli
Time [min]
+MS2 (16, 599.36), 7.4 min
1+
439.27
mV
(113)
TRA
(160)
1+
326.12
m/z
EIC 486.00 +All MS
mV-V-mV-TRA
21
Synthetic 21
21 in E. coli
Time [min]
+ MS2 (21, 486.26), 6.4 min
1+
326.17
mV
(113)
TRA
(160)
1+
(mV-V)213.04
1+
274.10
m/z
EIC 571.00 +All MS
V-V-V-mV-TRA
24
Synthetic 24
24 in E. coli
Time [min]
+ MS2 (24, 571.32), 6.9 min
1+
411.21 (V-V-V-mV)
TRA
(160)
m/z
EIC 585.00 +All MS
V-L-mV-V-TRA
25
Synthetic 25
25 from CabACD
Time [min]
1+
326.15
+ MS2 (25, 585.30), 7.1 min
mV
(113)
V
(99)
TRA
(160)
1+
425.20
1+
213.05 (V-L)
m/z

b

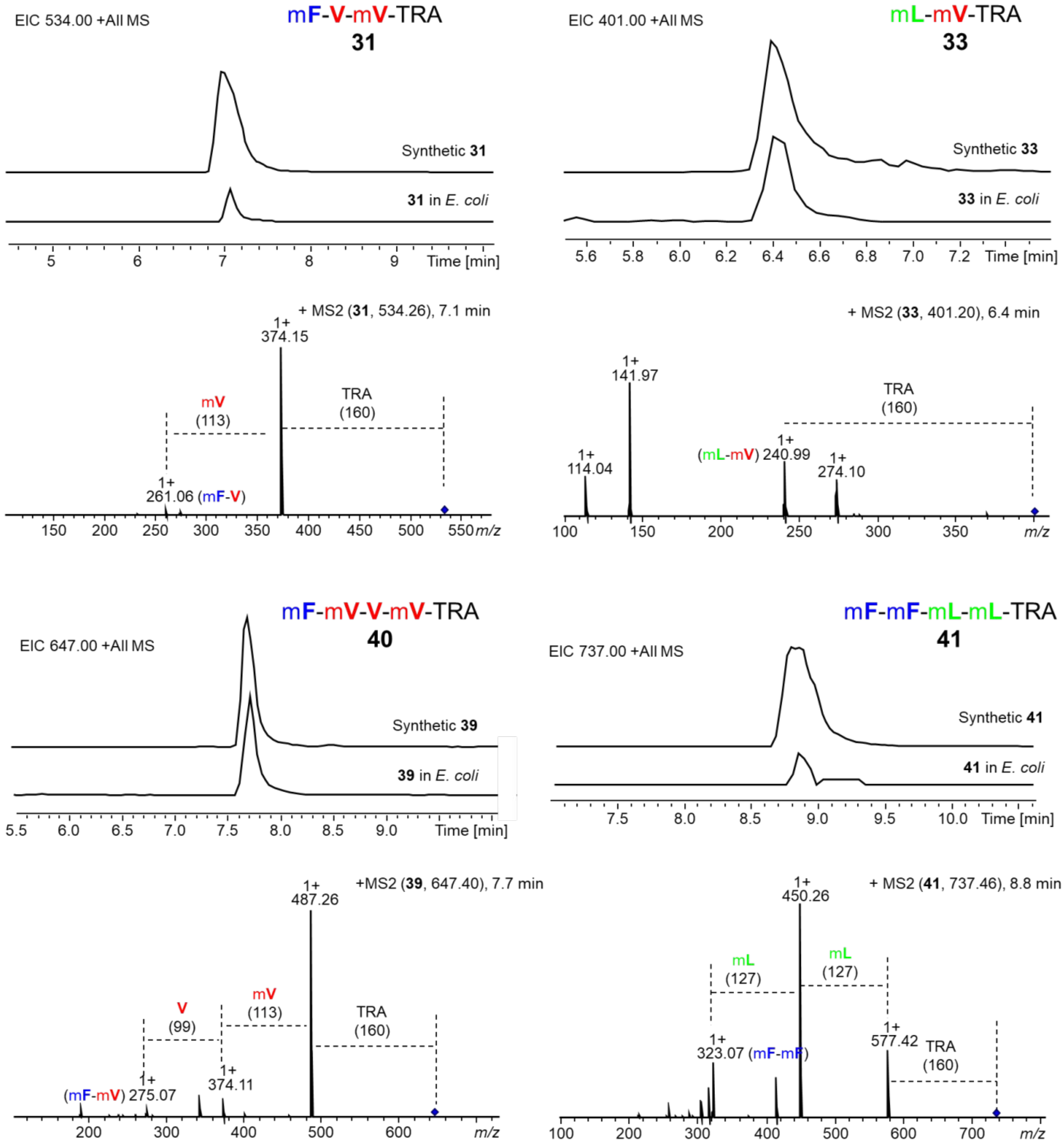

Figure S8. Final structural confirmation of major RXPs in selected constructs via comparison of HPLC-MS spectra between chemically synthesized RXPs and those produced in *E. coli* naturally.

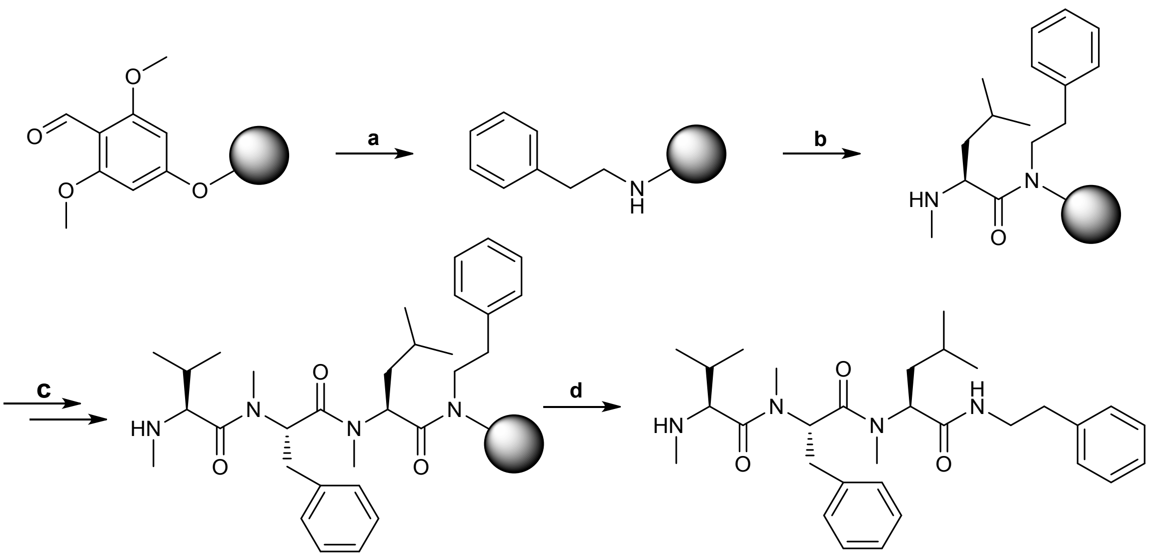

Figure S9. Synthesis of model mV-mF-mL-PEA (**13**). **a**, PEA (10 eq.), DMF/MeOH/AcOH (80:19:1), $NaBH_3CN$ (10 eq.), 60 °C, overnight. **b**, Fmoc-*N*-Me-Leu-OH (10 eq.), HATU (10 eq), HOAt (10 eq), DIPEA (20 eq.), DMF, overnight, then 20% piperidine/DMF. **c**, two steps, Fmoc-*N*-Me-Phe-OH (3 eq.), Fmoc-*N*-Val-OH (3 eq.), BTC (1.15 eq.), Collidine (10 eq.), DIPEA (8 eq.), THF, 2 h, then 20% piperidine/DMF. **d**, TFA/TIS/Water (95:2.5:2.5), 2 h.

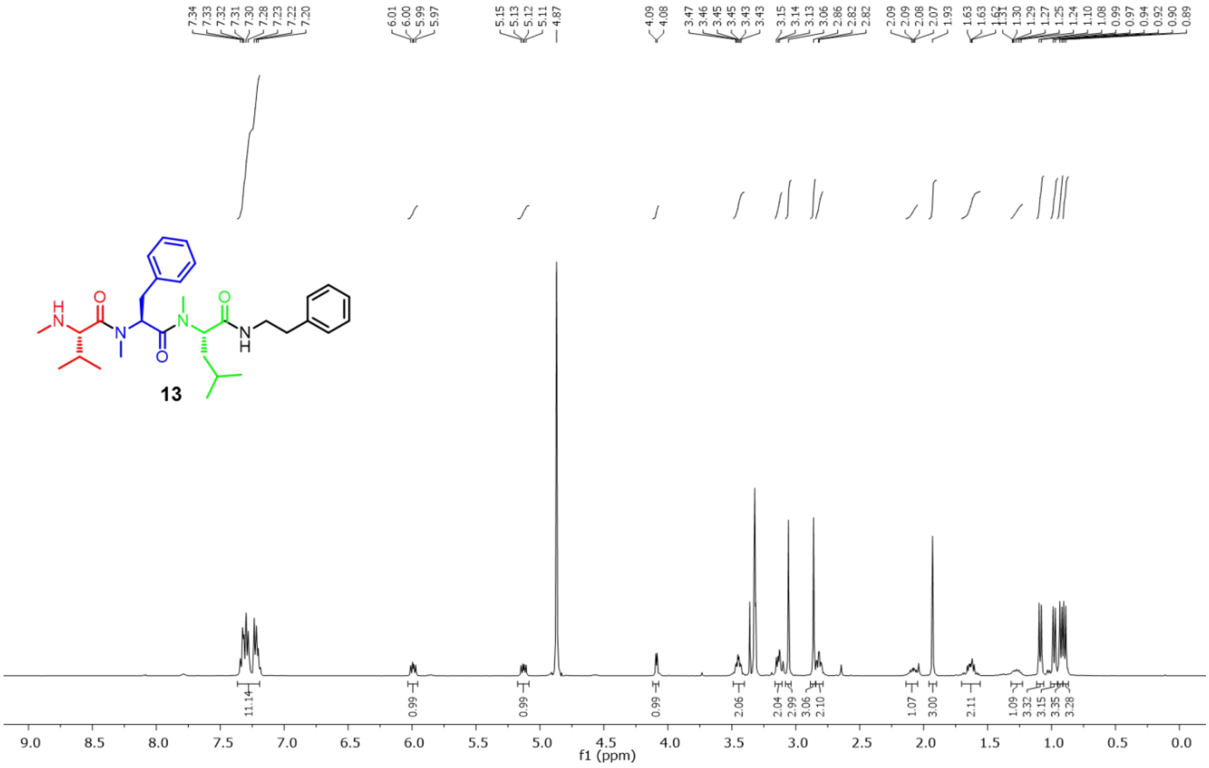

Figure S10. ^{1}H NMR spectrum of compound **13** (CD_3OD, 400 MHz).

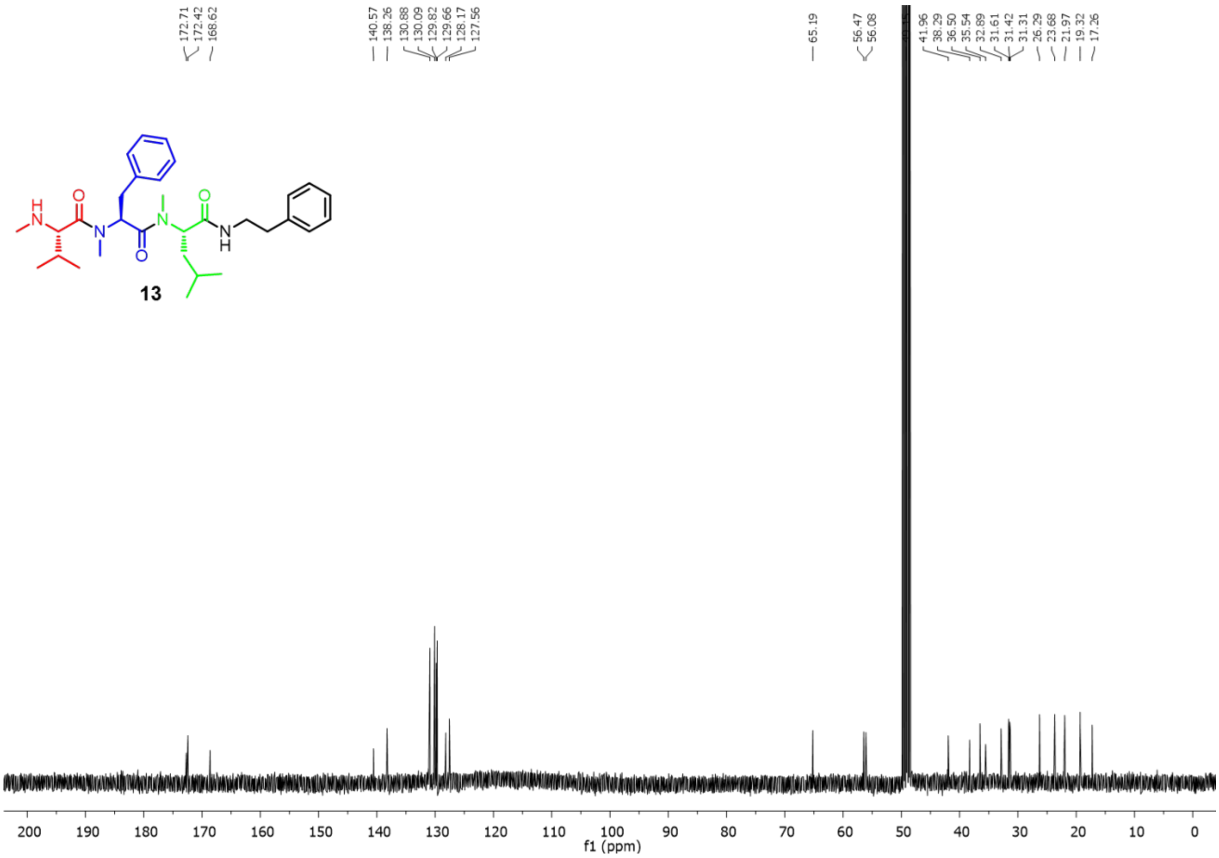

Figure S11. ^{13}C NMR spectrum of compound **13** (CD_3OD, 100 MHz).

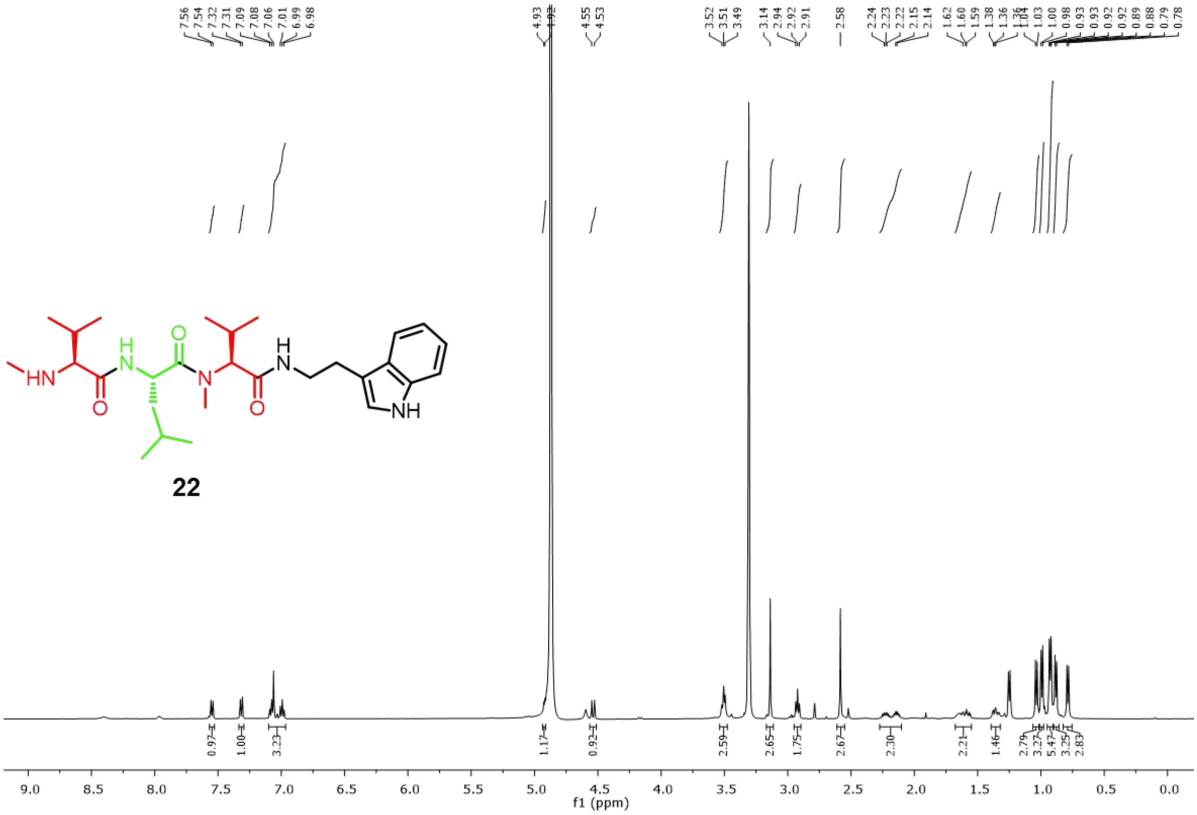

Figure S12. ^{1}H NMR spectrum of compound **22** (CD_3OD, 500 MHz).

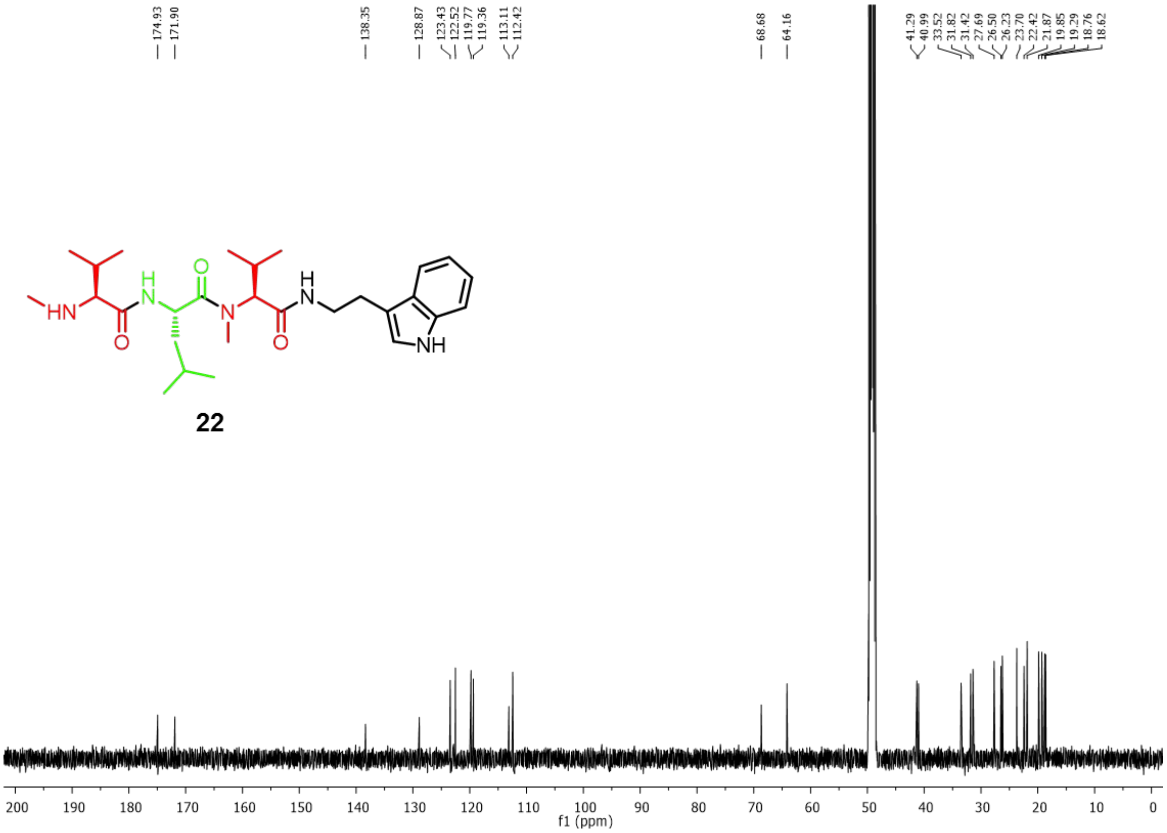

Figure S13. ^{13}C NMR spectrum of compound **22** (CD_3OD, 125 MHz).

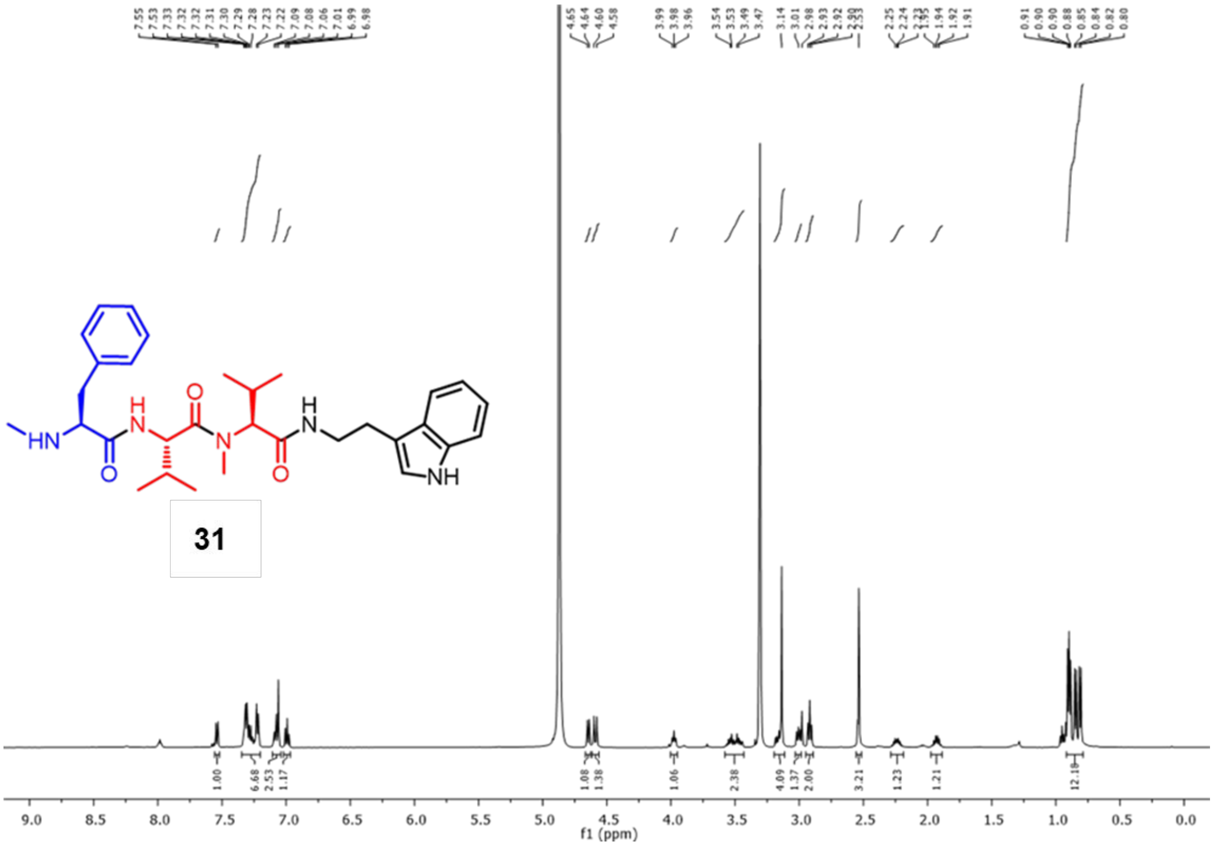

Figure S14. ^{1}H NMR spectrum of compound **31** (CD_3OD, 500 MHz).

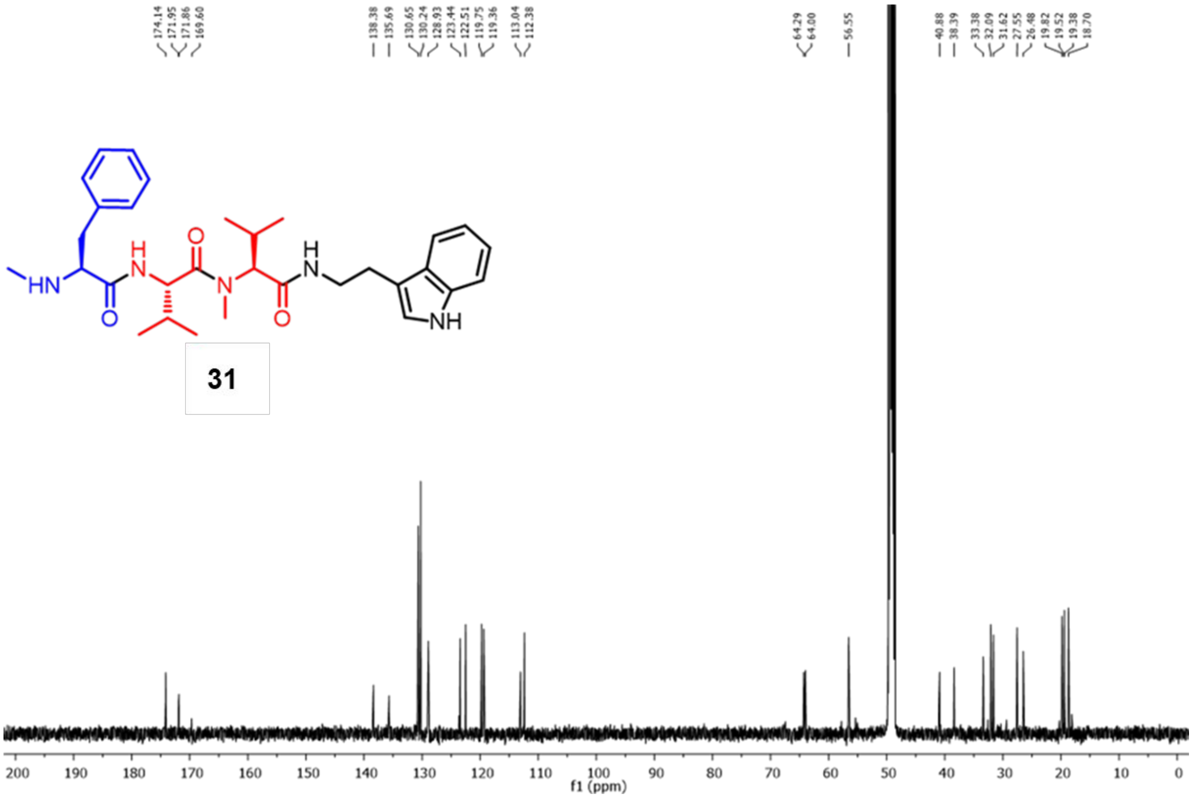

Figure S15. ^{13}C NMR spectrum of compound **31** (CD_3OD, 125 MHz).

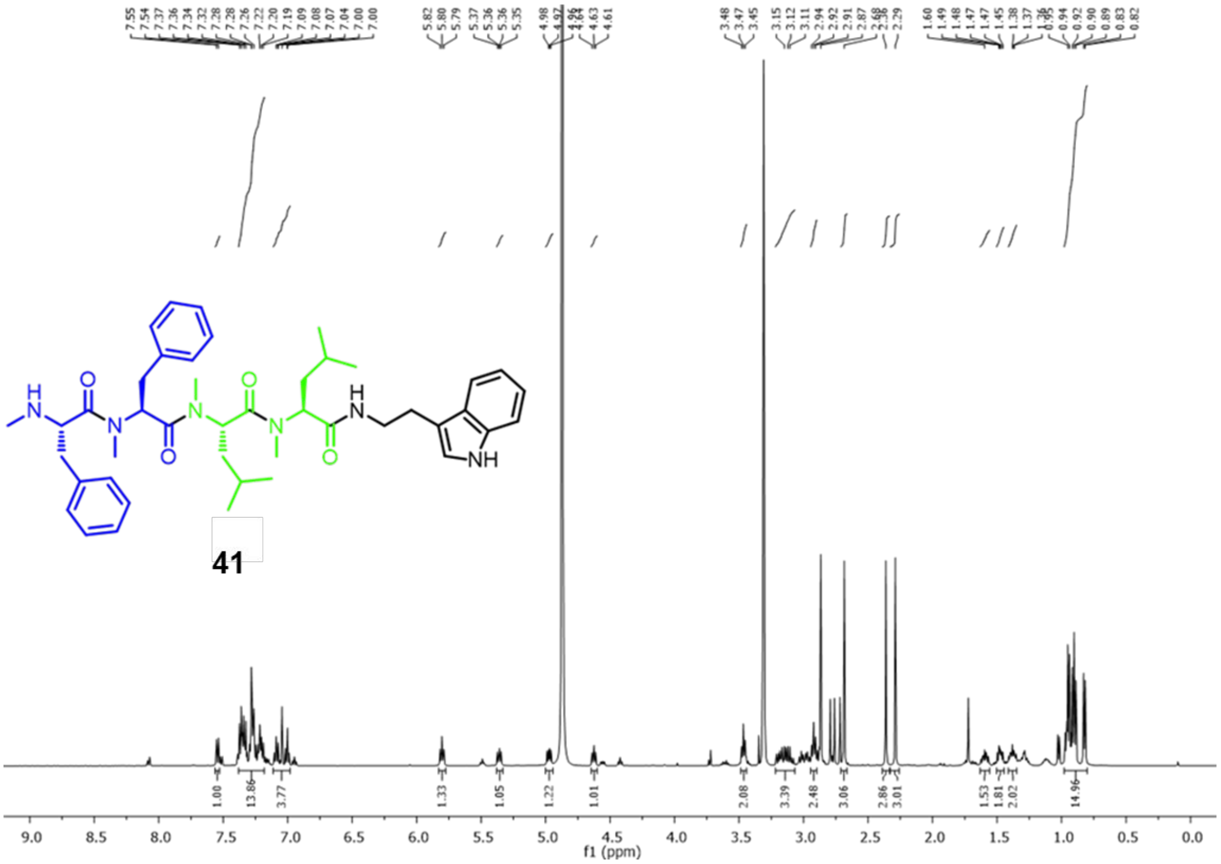

Figure S16. ^{1}H NMR spectrum of compound **41** (CD_3OD, 500 MHz).

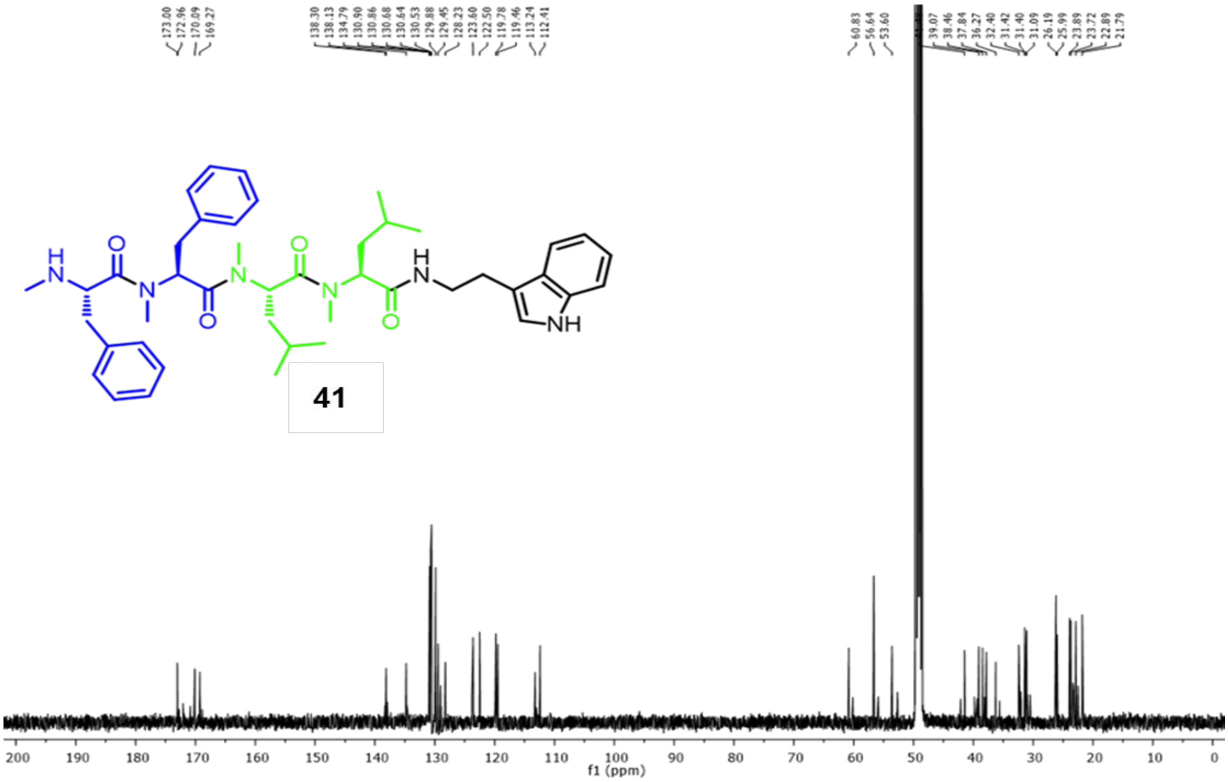

Figure S17. ^{13}C NMR spectrum of compound **41** (CD_3OD, 125 MHz).

References

(1) Cai, X., Nowak, S., Wesche, F., Bischoff, I., Kaiser, M., Fürst, R., and Bode, H. B. (2017) Entomopathogenic bacteria use multiple mechanisms for bioactive peptide library design. *Nat. Chem. 9*, 379–386.

(2) Schimming, O., Fleischhacker, F., Nollmann, F. I., and Bode, H. B. (2014) Yeast homologous recombination cloning leading to the novel peptides ambactin and xenolindicin. *Chembiochem 15*, 1290–1294.

(3) Thanwisai, A., Tandhavanant, S., Saiprom, N., Waterfield, N. R., Long, P. K., Bode, H. B., Peacock, S. J., and Chantratita, N. (2012) Diversity of *Xenorhabdus* and *Photorhabdus* spp. and their symbiotic entomopathogenic nematodes from Thailand. *PLoS One 7*, e43835.

(4) Reimer, D., Cowles, K. N., Proschak, A., Nollmann, F. I., Dowling, A. J., Kaiser, M., ffrench-Constant, R., Goodrich-Blair, H., and Bode, H. B. (2013) Rhabdopeptides as insect-specific virulence factors from entomopathogenic bacteria. *ChemBioChem 14*, 1991–1997.

(5) Hacker, C., Cai, X., Kegler, C., Zhao, L., Weickhmann, A. K., Wurm, J. P., Bode, H. B., and Wöhnert, J. (2018) Structure-based redesign of docking domain interactions modulates the product spectrum of a rhabdopeptide-synthesizing NRPS. *Nat. Commun. 9*, 4366.

(6) Cai, X., Zhao, L., and Bode, H. B. (2019) Reprogramming promiscuous nonribosomal peptide synthetases for production of specific peptides. *Org. Lett. 21*, 2116–2120.

(7) Stachelhaus, T., Mootz, H. D., and Marahiel, M. A. (1999) The specificity-conferring code of adenylation domains in nonribosomal peptide synthetases. *Chem. Biol. 6*, 493–505.

(8) Lombó, F., Velasco, A., Castro, A., de la Calle, F., Braña, A. F., Sánchez-Puelles, J. M., Méndez, C., and Salas, J. A. (2006) Deciphering the biosynthesis pathway of the antitumor thiocoraline from a marine actinomycete and its expression in two *Streptomyces* Species. *ChemBioChem 7*, 366–376.

(9) Tillett, D., Dittmann, E., Erhard, M., von Döhren, H., Börner, T., and Neilan, B. A. (2000) Structural organization of microcystin biosynthesis in microcystis aeruginosa PCC7806: an integrated peptide–polyketide synthetase system. *Chem. Biol. 7*, 753–764.

(10) Nishizawa, T., Ueda, A., Nakano, T., Nishizawa, A., Miura, T., Asayama, M., Fujii, K., Harada, K., and Shirai, M. (2011) Characterization of the locus of genes encoding enzymes producing heptadepsipeptide micropeptin in the unicellular cyanobacterium microcystis. *J. Biochem. 149*, 475–485.

(11) Labby, K. J., Watsula, S. G., and Garneau-Tsodikova, S. (2015) Interrupted adenylation domains: unique bifunctional enzymes involved in nonribosomal peptide biosynthesis. *Nat. Prod. Rep. 32*, 641–653.
